# ACMSD inhibition corrects fibrosis, inflammation, and DNA damage in MASLD/MASH

**DOI:** 10.1016/j.jhep.2024.08.009

**Published:** 2024-08-22

**Authors:** Yasmine J. Liu, Masaki Kimura, Xiaoxu Li, Jonathan Sulc, Qi Wang, Sandra Rodríguez-López, Angelique M.L. Scantlebery, Keno Strotjohann, Hector Gallart-Ayala, Archana Vijayakumar, Robert P. Myers, Julijana Ivanisevic, Riekelt H. Houtkooper, G. Mani Subramanian, Takanori Takebe, Johan Auwerx

**Affiliations:** 1 Laboratory of Integrative Systems Physiology, Institute of Bioengineering, École Polytechnique Fédérale de Lausanne, 1015 Lausanne, Switzerland; 2 Division of Gastroenterology, Hepatology and Nutrition & Division of Developmental Biology, Cincinnati Children’s Hospital Medical Center, Cincinnati, OH 45229, USA; 3 OrsoBio, Inc., Menlo Park, CA, USA; 4 Laboratory Genetic Metabolic Diseases, Amsterdam UMC, Amsterdam, The Netherlands; 5 Amsterdam Gastroenterology, Endocrinology and Metabolism institute, Amsterdam UMC, Amsterdam, The Netherlands; 6 Amsterdam Cardiovascular Sciences institute, Amsterdam UMC, Amsterdam, The Netherlands; 7 Premium Research Institute for Human Metaverse Medicine (WPI-PRIMe), and Division of Stem Cell and Organoid Medicine, Osaka University, Suita, Osaka 565-0871, Japan; 8 Metabolomics Platform, Faculty of Biology and Medicine, University of Lausanne, Lausanne, Switzerland

**Keywords:** NAD^+^, ACMSD, DNA repair, MASLD/MASH, Mendelian randomization, human liver organoids

## Abstract

**Background & Aims:**

Recent findings reveal the importance of tryptophan-initiated *de novo* nicotinamide adenine dinucleotide (NAD^+^) synthesis in the liver, a process previously considered secondary to biosynthesis from nicotinamide. The enzyme α-amino-β-carboxymuconate-ε-semialdehyde decarboxylase (ACMSD), primarily expressed in the liver and kidney, acts as a modulator of *de novo* NAD^+^ synthesis. Boosting NAD^+^ levels has previously demonstrated remarkable metabolic benefits in mouse models. In this study, we aimed to investigate the therapeutic implications of ACMSD inhibition in the treatment of metabolic dysfunction-associated steatotic liver disease/steatohepatitis (MASLD/MASH).

**Methods:**

*In vitro* experiments were conducted in primary rodent hepatocytes, Huh7 human liver carcinoma cells and induced pluripotent stem cell-derived human liver organoids (HLOs). C57BL/6J male mice were fed a western-style diet and housed at thermoneutrality to recapitulate key aspects of MASLD/MASH. Pharmacological ACMSD inhibition was given therapeutically, following disease onset. HLO models of steatohepatitis were used to assess the DNA damage responses to ACMSD inhibition in human contexts.

**Results:**

Inhibiting ACMSD with a novel specific pharmacological inhibitor promotes *de novo* NAD^+^ synthesis and reduces DNA damage *ex vivo*, *in vivo*, and in HLO models. In mouse models of MASLD/MASH, *de novo* NAD^+^ biosynthesis is suppressed, and transcriptomic DNA damage signatures correlate with disease severity; in humans, Mendelian randomization-based genetic analysis suggests a notable impact of genomic stress on liver disease susceptibility. Therapeutic inhibition of ACMSD in mice increases liver NAD^+^ and reverses MASLD/MASH, mitigating fibrosis, inflammation, and DNA damage, as observed in HLO models of steatohepatitis.

**Conclusions:**

Our findings highlight the benefits of ACMSD inhibition in enhancing hepatic NAD^+^ levels and enabling genomic protection, underscoring its therapeutic potential in MASLD/MASH.

## Graphical Abstract

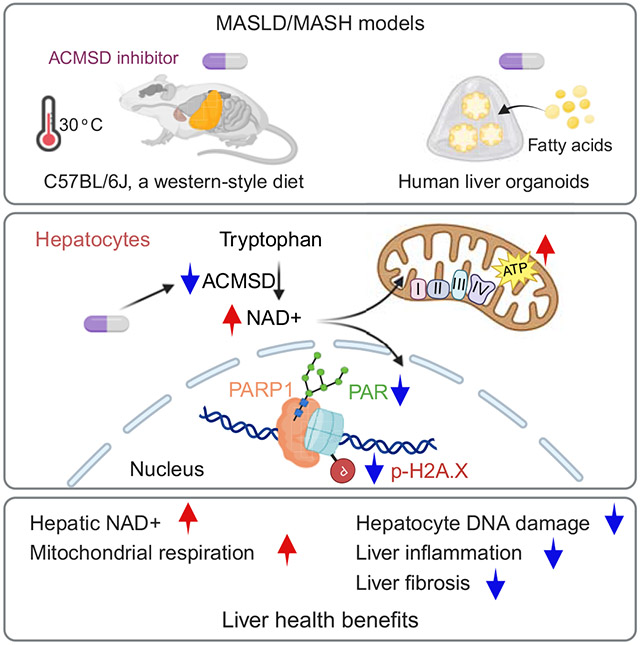

## Introduction

Metabolic dysfunction-associated steatotic liver disease (MASLD), previously known as non-alcoholic fatty liver disease, is a global health challenge, with its onset and progression shaped by genetic, epigenetic, and environmental factors.^1,2^ MASLD is estimated to afflict up to 25% of the global population and its severe form, metabolic dysfunction-associated steatohepatitis (MASH), characterized by lobular inflammation and hepatocyte ballooning, can progress to cirrhosis, an advanced form of liver fibrosis and a potentially fatal hepatic condition.^3,4^ Mitochondrial dysfunction,^5,6^ DNA damage,^7-10^ endoplasmic reticulum stress,^11^ oxidative stress,^10,12^ loss of proteostasis,^13^ and accumulation of cytotoxic lipid intermediates^14^ have all been described as drivers of MASLD/MASH, but their relative contribution to the molecular underpinning of the disease remains debated.

Nicotinamide adenine dinucleotide (NAD^+^) is a ubiquitous electron acceptor in glycolysis and the Krebs cycle, while NADH serves as the primary electron donor in the mitochondrial respiratory chain.^15,16^ Furthermore, NAD^+^ is also a co-substrate for non-redox enzymes, including poly (ADP-ribose) polymerases (PARPs), sirtuins, and ectonucleotidases, affecting essential processes such as metabolic fitness, DNA damage repair, and immune and inflammatory processes.^17^ Beyond salvaging NAD^+^ precursors, hepatocytes can synthesize NAD^+^ *de novo* from tryptophan, which is pivotal to sustaining the hepatic NAD^+^ reservoir.^17-19^ In *de novo* NAD^+^ synthesis, α-amino-β-carboxymuconate-ε-semialdehyde decarboxylase (ACMSD) catalyzes a branching point, directing the intermediate ACMS towards complete oxidation to acetyl-CoA instead of NAD^+^ synthesis (Fig. S1A). ACMSD is mainly expressed in the kidney and liver,^20^ where the enzyme acts as a modulator of *de novo* NAD^+^ production. This effect is especially pronounced in the liver, which in mice assimilates 9-fold more tryptophan for this purpose than the kidney.^19^

We have previously demonstrated that inhibiting ACMSD can increase NAD^+^ levels in mouse liver.^18^ Nonetheless, its therapeutic potential in mouse models that mimic human MASLD/MASH is unexplored. Here, we revealed that inhibiting ACMSD in hepatocytes is cytoprotective, largely through genome-protective effects. Correlation analysis linked liver transcriptome signatures of DNA damage to liver disease phenotypes, while genetic analysis via Mendelian randomization showed significant effects of DNA damage on plasma alanine aminotransferase (ALT) levels. These results suggest a causal role of liver DNA damage in MASLD/MASH. We then demonstrated the therapeutic efficacy of a novel ACMSD inhibitor in a dietary mouse model of MASLD/MASH, through the mitigation of DNA damage, and confirmed the translational relevance of ACMSD inhibition in human liver organoid (HLO) models of steatohepatitis.

## Materials and methods

### Primary hepatocyte culture

Mouse primary hepatocytes (mPHs) were isolated from 8- to 12-week-old C57BL/6J or *Acmsd*^L2/L2^ mice in a mixed background of C57BL/6J and C57BL/6N (males and females) using the liberase perfusion method as previously described,^41^ with minor modifications. mPHs were cultured with DMEM medium (Gibco) including 10% FBS (Gibco), 10 U/ml penicillin, 0.5 mM tryptophan (T0254, Sigma-Aldrich), MEM non-essential amino acids (Gibco) and HEPES (Gibco). Following attachment, the original medium was replaced with serum-free media supplemented with TLC-065 (US patent no. PCT/US17/68673), or DMSO. mPHs extracted from *Acmsd*^L2/L2^ mice were transduced either with adenovirus encoding CRE recombinase (to generate *Acmsd* knockout [KO], Ad-iCre, #1045 Vector Biolabs) or adenovirus encoding GFP/RFP (Ad-eGFP, #1060; Ad-RFP, #1660; Vector Biolabs) at multiplicities of infection (MOI) = 5. mPHs were collected after 24h (TLC-065 studies) unless otherwise indicated, or 48h (adenoviral studies). For studies evaluating effects of TLC-065 in *Acmsd* KO mPHs, *Acmsd*^L2/L2^ mPHs were transduced as described above for 24 h to generate *Acmsd* wild-type (WT) and *Acmsd* KO, and then treated with TLC-065 or DMSO for an additional 24 h before harvesting. When indicated, cell culture medium was supplemented with palmitate-BSA (Sodium palmitate, Sigma-Aldrich, #P9767-5G) or Doxorubicin (BP990, Sigma).

### Cell culture

HuH7 cells were grown at 37 °C in a humidified atmosphere of 5% CO_2_/95% air in RPMI 1640 medium (Gibco) supplemented with 10% FBS.

### Enzymatic cyclic assay for quantitative NAD^+^ determination

To extract NAD^+^, cells were rapidly cooled using 300 μl of 2 M perchloric acid (HClO_4_) and then transferred into 1.5 ml tubes. The mixture was spun at 16,000 g for 5 min. 100 μl of the acidic supernatant was then taken and neutralized with the addition of 150 μl of a 2 M/0.6 M solution of KOH/MOPS. The quantification of NAD^+^ was performed using a spectrophotometric enzymatic cycling assay using previously described methods.^42^

### Apoptosis assessment

Caspase 3/7 activities were measured with the Caspase-Glo assay (#G8091, Promega) according to the manufacturer’s instructions. Apoptosis in mPHs was induced by exposing cells to 0.75 mM palmitate for 36 h or 1 μM doxorubicin for 24 h.

### Oxygen consumption

Oxygen consumption was measured by Seahorse XF96 analyzer (from Seahorse Bioscience). mPHs were plated in collagen-coated (Sigma-Aldrich C3867) seahorse XF96 cell culture microplates at 10,000 cells/well. To induce maximal respiration, the uncoupling agent FCCP was applied at a concentration of 2 μM and injected twice.

### Fatty acid oxidation

100,000 HuH-7 cells were plated in 24-well plate with 500 μl DMEM (Gibco) and incubated in a humidified incubator with 5% CO_2_ at 37 °C overnight. After overnight incubation, the medium was refreshed, TLC-065 was added to the cell culture at the specified concentrations and maintained for 30 min at 37 °C cell incubator. 4 μl of ^14^C-palmitate (#NEC075H050UC, Perkin Elmer) was added to the cell culture for a 16 h incubation. Following this, 400 μl of supernatant was transferred to 1.5 ml Eppendorf tubes and 40 μl of 10% BSA (fatty acid free, #A3803, Sigma) and 40 μl of 60% HClO_4_ (#10015161, Sinopharm chemical reagent) were added. This mixture was vortexed for 5 min and then spun at 14,000 rpm for 5 min. 350 μl of supernatant was then transferred to 4 ml of Microbeta vial (PerkinElmer CS/3000) with 2 ml UltimaGold (#6013329, PerkinElmer), incubated for 10 min at room temperature and read by MicroBeta (#2450, PerkinElmer). HuH-7 cells were purchased from JCRB (JCRB0403).

### *De novo* lipogenesis

Primary rat hepatocytes (BioIVT, M00065) cultured in William’s E medium (Gibco, A1217601) were incubated with a dose range of TLC-065 diluted in DMSO. *De novo* lipogenesis was measured by incubating rat primary cells with 2 μCi ^13^C-labelled acetate (#NEC085H001MC, PerkinElmer) ± different doses of TLC-065 for 4 h and examining incorporation into cellular lipids with scintillation counting by MicroBeta (PerkinElmer, 2450).

### Reactive oxygen species quantification

Cells were stained with 20 μM 2’,7’-dichlorofluorescin reagent (ab113851, Abcam) for 45 to 60 min at 37 °C in the dark. After staining, cells were rinsed twice using PBS. An iD3 multi-mode microplate reader was used for signal quantification using an excitation and emission wavelength of 485 nm and 535 nm, respectively.

### RNA isolation for RNA-seq and RT-PCR

RNA of tissue and cell pellets were extracted from TRIZOL (Invitrogen) and purified using the NucleoSpin RNA kit (MACHEREY-NAGEL) following the manufacturer’s instructions. For RNA-seq, samples underwent quality checks for purity and fragmentation (Fragment Analyzer).

cDNA was generated from reverse transcription using QuantiTect Rev. Transcription Kit (Qiagen) and analyzed by reverse-transcription quantitative PCR (SYBR Greem chemistry) using the Light-Cycler system (Roche Applied Science). The primers are listed in Table S8 and supplementary CTAT table.

### Protein isolation and western blot

Proteins were extracted using RIPA buffer containing protease inhibitor (#78430, Thermo Fisher Scientific) and phosphatase inhibitor (#78428, Thermo Fisher Scientific). The concentration of extracted protein was then determined and normalized using the DC Protein Assay Reagents (#5000116, Bio-Rad). Subsequently, the lysates were analyzed by SDS–PAGE and western blot. Proteins were detected using the following antibodies: p-H2A.X (#2577, CST, 1:1000), H2A.X (#sc-54607, Santa Cruz, 1:200), HSP90 (#sc-101494, Santa Cruz, 1:500), Cleaved caspase-3 (#9661S, CST, 1:1000), pro-caspase-3 (#9662S, CST, 1:1000), VINCULIN (#ab129002, Abcam, 1:1000), p-IκBα (#9246S, CST, 1:1000), IκBα (L35A5) (#4814, CST, 1:1000), Poly-ADP-ribose (#ALX-804-220-R100, ENZO life sciences, 1:1000), PARP (46D11) (#9532, CST, 1:1000).

### Immunoprecipitation from liver lysates

PARP1 was immunoprecipitated from mouse liver lysates using Dynabeads^™^ Protein A Kit (#10006D, Invitrogen). Each sample contained 30 mg of liver powder homogenized in 300 μl of Pierce^™^ IP Lysis Buffer (#87787) with 0.25% sodium deoxycholate, 10 μM PARGi, 10 μM Olaparib, and protease and phosphatase inhibitors. After sonication and centrifugation, the supernatant (>1 mg protein) was incubated with a PARP1 antibody-bead complex at 4 °C for 2 h, washed three times, and eluted for western blot analysis of PARP1 and PAR. PARP (46D11) (#9532, CST) and Rabbit (DA1E) mAb IgG XP^®^ Isotype Control (#3900, CST).

### Measurements of NAD^+^ metabolome

Measurements of NAD^+^ metabolome from mPHs (~4.0 × 10^5^ cells) and tissue samples (30-40 mg) were performed by liquid chromatography–tandem mass spectrometry analysis as described elsewhere^43^ and in the supplementary materials and methods.

### A dietary mouse model of MASLD/MASH

Male C57BL/6J mice were obtained from Charles River and acclimatized for 2 weeks at the EPFL animal facility before being divided into three groups. One group received a western diet (WD; Research Diets D12079B; 40% calories from fat, 17% from protein, 43% from carbohydrates), another received the same WD diet admixed with 25 mg/kg body weight/day of TLC-065 (US patent PCT/US17/68673), and the third was fed a control diet (CD; Research Diets D16042904B; 10% calories from fat, 17% from protein, 73% from carbohydrates). Starting from 7 weeks, all mice were kept at 30 °C (thermoneutrality [TN]). TLC-065 treatment began at 16 weeks. Body weight was monitored biweekly. All procedures were conducted in compliance with Swiss ethical standards and approved by the Service de la Consommation et des Affaires Vétérinaires of the Canton de Vaud (license VD3313.1).

At 30 weeks of age, mice fasted for 4 h in the morning were subjected to isoflurane anesthesia between 1:30 pm to 4.00 pm. The blood was sampled via cardiac puncture, placed into EDTA-coated tubes and centrifuged at 4,500 rpm for 10 min at 4 °C. The plasma supernatant was collected and flash-frozen in liquid nitrogen for plasma analyses. Collected tissues were snapped frozen in liquid nitrogen. Parts of liver were stored in formalin or optimal cutting temperature compound for histological analysis.

### Histopathology on liver tissues of WD/TN mice

Liver samples were taken from the same lobe of each animal and were fixed in buffered formalin (4%) overnight and embedded in paraffin. 4 μm serial paraffin sections were made from paraffin embedded liver then stained with standard H&E to assess the general morphology, and Sirius red for collagen fiber content. Detection of immune cells (rat α-CD45, clone 30F-11, Thermo Fisher 14-0451-82, diluted 1:200) or DNA damage (rabbit anti phospho-H2AX [ser139], CST #2577, diluted 1:100) was performed using the fully automated Ventana Discovery ULTRA (Roche Diagnostics, Rotkreuz, Switzerland). All steps were performed on the equipment with Ventana solutions. Briefly, dewaxed and rehydrated paraffin sections were pretreated with heat for 40 min and CC1 solution. The primary antibodies were incubated for 1 h at 37 °C. After incubation with rat or rabbit ImmPRESS HRP (Ready to use, Vector laboratories Laboratories), chromogenic revelation was performed with ChromoMap DAB kit (Roche Diagnostics, Rotkreuz, Switzerland). Sections were counterstained with Harris hematoxylin and permanently mounted. 8 μm cryosections were stained with Oil Red O and cytochrome c oxidase activity as previously described.^6^

Images were taken with an Olympus Slide Scanner VS120 L100 at 20x magnification. Digital slides were analyzed using QuPath software. For the number of CD45^+^ cells and quantification of Oil Red O^+^ area, the whole stained liver section was quantified. For Sirius red^+^ area, 6-8 random fields were quantified on each slide from individual mouse. For p-H2A.X, 4-6 random fields were quantified on each slide from individual mice.

### Human MASLD/MASH RNA-seq datasets

Public human liver bulk RNA-seq processed counts were downloaded from the GEO (Gene Expression Omnibus) under the accession numbers GSE135251,^25^ GSE162694,^26^ and GSE130970.^27^ The samples (participants) were grouped based on the stage of fibrosis and analysis was described in the supplementary materials and methods.

### HLO models of steatohepatitis

GCKR^CC^ and GCKR^TT^ human liver organoids (HLOs) were generated from the induced pluripotent stem cell line iPSC_72.3 (from CCHMC Pluripotent Stem Cell Facility), CR00012 (from National Institute of Neurological Disorders and Stroke Human Cell and Data Repository) and 1383D6 (CiRA, Center for iPS Cell Research and Application), using the previously described method.^30^ For quantification of lipid accumulation, steatohepatitic HLOs (sHLOs) were stained with BODIPY 493/503 (ThermoFisher Scientific) and NucBlue Live ReadyProbes Reagent (ThermoFisher Scientific). Next, sHLOs were imaged with a Nikon A1 inverted confocal microscope (Japan) and a Keyence BZ-X710 automated fluorescence microscope (Japan). The volume of lipid droplets was quantified through the Analysis Application Hybrid cell count (Keyence) and was subsequently normalized against the signal from each nucleus.

### DNA damage induction and detection in HLOs

To induce DNA damage with palmitate, HLOs isolated from Matrigel were cultured with 300 μM sodium palmitate (#P9767 Sigma) for 48h. To induce DNA damage with doxorubicin, HLOs isolated from Matrigel were cultured with 5 μM doxorubicin hydrochloride (#D1515, Millipore-Sigma) for 24 h. The treated HLOs were fixed with 4% PFA, and whole-mount staining was performed as previously described.^44^ DNA damage was detected using Phospho-53BP1 (Ser1778) Antibody (#2675 CST) and p-H2A.X (ser139, #ab26350, Abcam). Stained HLOs were imaged with a Nikon A1 inverted confocal microscope (Japan) and a Keyence BZ-X710 automated fluorescence microscope (Japan). DNA damage was quantified by Imaris Cell Imaging Software and then normalized against the signal from each nucleus.

### Quantification and statistical analysis

Detailed information about the value of n, along with significance and error bars, are provided in the legends of each figure. All the replicates are biological replicates. For data presented in a box-whisker plot, the box extends from the 25th to 75th percentile with the median shown as a line in the middle, and whiskers indicate smallest and largest values. A two-tailed Student’s *t* test was used to calculate statistical differences between the means of two groups. One-way or two-way ANOVA tests were used for multiple group comparisons. ANCOVA was used to analyze locomotor activity and energy expenditure. Statistical analyses were conducted using R. The threshold for statistical significance was set at *p* values less than 0.05; *p <0.05; **p <0.01; ***p <0.001.

### Permissions to human databases

This research has been conducted using the UK Biobank Resource under Application Number 48020 and GTEx, dbGaP accession number phs000424.v8 under project number 10143.

## Results

### Inhibiting ACMSD enhances the NAD^+^ metabolome and bioenergetics in hepatocytes

**Fig. 1.**
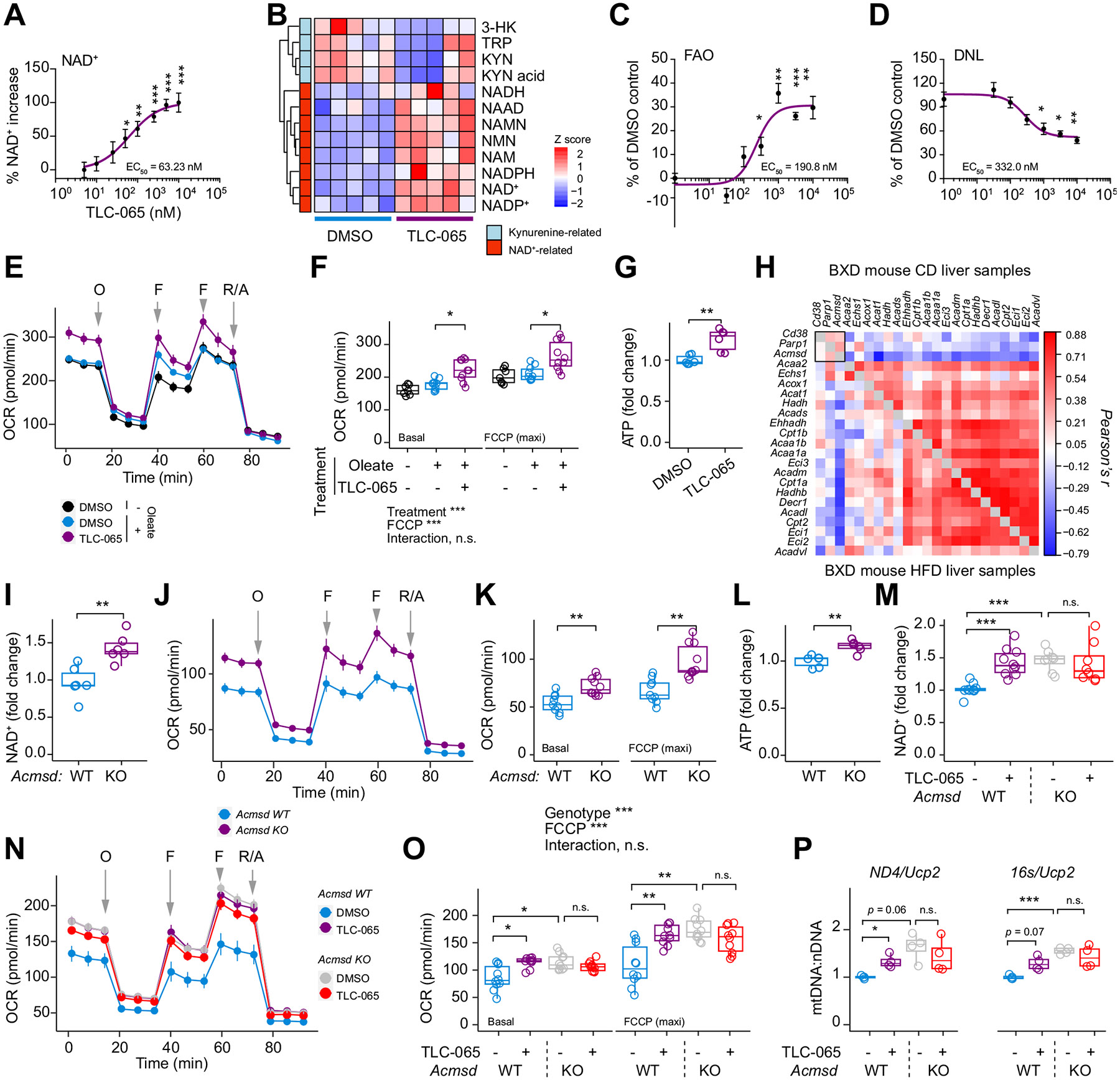
ACMSD inhibition enhances the NAD+ metabolome and mitochondrial respiration. (A) Dose-response increase of NAD^+^ in mPHs after 24 h TLC-065 exposure (n = 4). (B) Targeted metabolomics in mPHs exposed to 0.5 μM TLC-065 for 24 h (n = 5). (C) Dose-response curve of FAO in HuH-7 cells after 16 h TLC-065 exposure (n = 3). (D) Dose-response curve of DNL in primary rat hepatocytes after 4 h TLC-065 exposure (n = 3). (E-F) Time course and boxplot of mitochondrial respiration in mPHs (n = 7 control; n = 10 other groups, 0.1 μM TLC-065 for 24 h). (G) ATP levels in mPHs (n = 5, 0.5 μM TLC-065 for 24 h). (H) Pearson correlation of liver transcripts of FAO genes and *Acmsd* in BXD mice fed either chow or HFD. (I-L) NAD^+^ fold changes (n = 6) (I), mitochondrial respiration (n = 9 WT; 10 *Acmsd* KO) (J-K), and ATP fold changes (n = 6) (L) in *Acmsd* WT or KO mPHs. (M-P) NAD^+^ fold changes (n = 10 WT; 8 KO) (M), mitochondrial respiration (n = 10) (N–O), and mtDNA:nDNA (n = 4) (P) in *Acmsd* WT or KO mPHs exposed to 0.1 μM TLC-065 for 24 h. Error bar: mean ± SEM. *p <0.05; **p <0.01; ***p <0.001, two-sided Student’s *t* test (A, C-D, G, I, L). Two-way ANOVA and Bonferroni multiple comparisons test (F, K, M, O–P). DNL, *de novo* lipogenesis; FAO, fatty acid beta-oxidation; F, FCCP (carbonyl cyanide-p-trifluoromethoxyphenylhydrazone); HFD, high-fat diet; KO, knockout; mPHs, mouse primary hepatocytes; mtDNA:nDNA, mitochondrial/nuclear DNA; O, oligomycin; R/A, rotenone/antimycin; WT, wild-type. 3-HK, 3-OH kynurenine; TRP, tryptophan; KYN, kynurenine; NAD^+^, nicotinamide adenine dinucleotide; NADH, nicotinamide adenine dinucleotide hydrogen; NAAD, nicotinic acid adenine dinucleotide; NMN, nicotinamide mononucleotide; NAMN, nicotinic acid mononucleotide; NAM, nicotinamide; NADP(H), nicotinamide adenine dinucleotide phosphate (hydrogen).

In mouse primary hepatocytes (mPHs), TLC-065, a novel ACMSD-specific pharmacological inhibitor (US patent no. PCT/US17/68673), dose-dependently boosted NAD^+^ levels (EC_50_ = 63.23 nM) over 24 h (Fig. 1A). Targeted metabolomics in mPHs treated with 0.5 μM TLC-065 for 24 h significantly increased NAD^+^ levels by 1.6-fold and metabolites in the salvage pathway (Fig. 1B and Fig. S1A, B). In contrast, TLC-065 did not affect the NAD^+^/NADH ratio or the levels of metabolites upstream of ACMSD, such as kynurenine (Fig. 1B and Fig. S1B, C).

TLC-065 further increased fatty acid oxidation (FAO) (EC_50_ = 190.8 nM) in the HuH-7 human liver carcinoma cell line (Fig. 1C). This was accompanied by a dose-dependent reduction in *de novo* lipogenesis (EC_50_ = 332.0 nM) as measured in rat primary hepatocytes (Fig. 1D). TLC-065 also increased basal and maximal oxygen consumption rate, ATP concentration, and the mitochondrial:nuclear DNA ratio in mPHs and HuH-7 cells (Fig. 1E-G and Fig. S1D, E). In line with enhanced FAO by ACMSD inhibition, *Acmsd* expression negatively correlated with the expression of FAO genes in the livers of genetically diverse BXD strains^21^ fed either chow or high-fat diets (Fig. 1H).

Deletion of ACMSD using adenoviral Cre recombinase in *ACMSD*^lox/lox^ mPHs resulted in a >96% reduction in *Acmsd* expression (Fig. S1F). Further, *Acmsd* KO mPHs had higher NAD^+^ content, oxygen consumption rate, and ATP levels compared to the WT controls (Fig. 1I-L). TLC-065 treatment of *Acmsd* KO mPHs did not cause additional increases in these parameters (Fig. 1M-P and Fig. S1G,H), confirming that the effects of TLC-065 are mediated via inhibition of ACMSD.

### Inhibiting ACMSD mitigates DNA damage and innate immune responses in hepatocytes

**Fig. 2.**
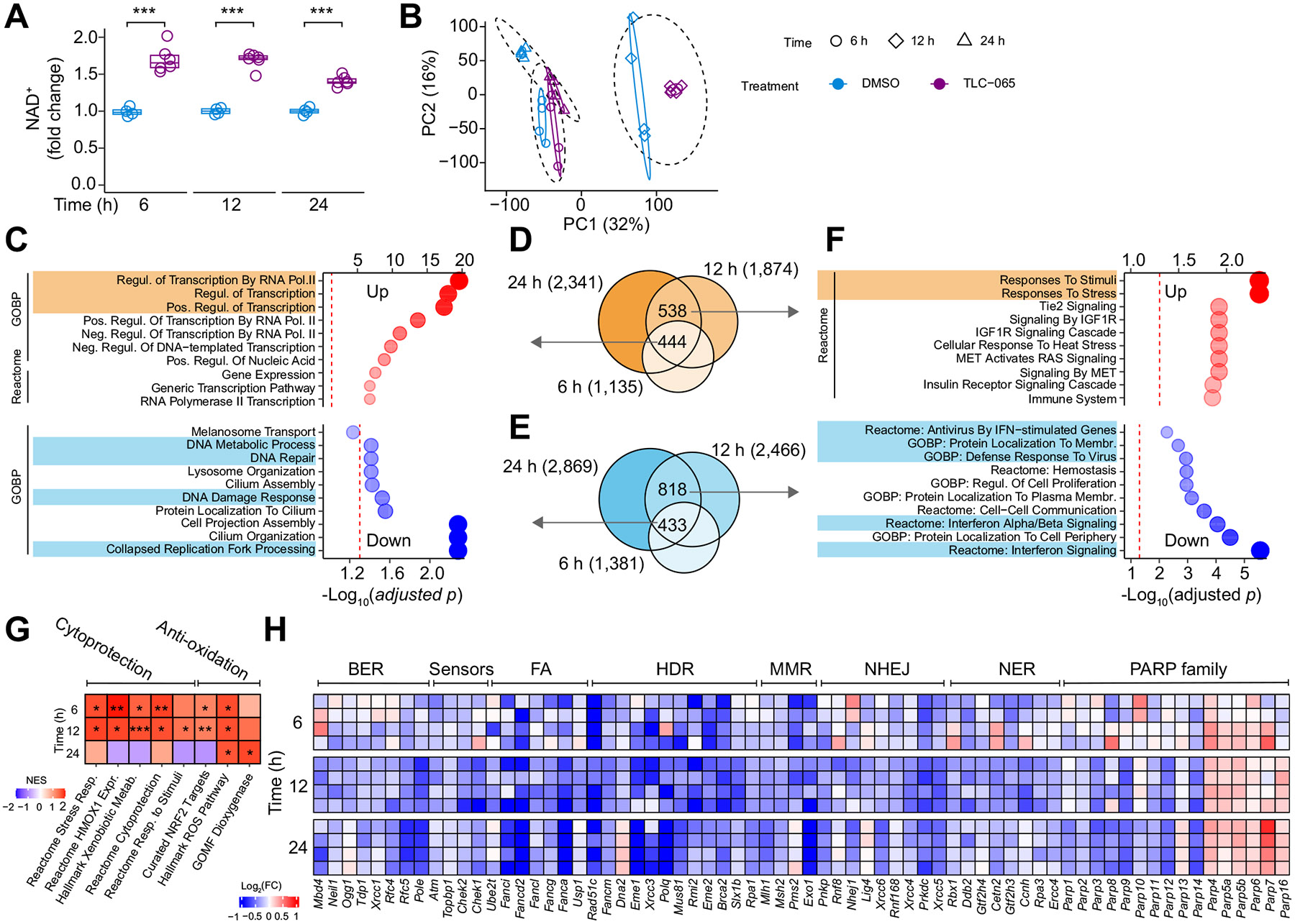
ACMSD inhibition downregulates the DNA damage and interferon response in hepatocytes. (A) Fold changes of NAD+ in mPHs exposed to 0.5 μM TLC-065 (n = 6). (B) PCA of normalized expression of mPHs exposed to 0.5 μM TLC-065 (n = 4). (C) Top10 up- or downregulated gene sets overlapping at three timepoints. (D-E) Venn diagram displaying the number of overlapping upregulated (D) and downregulated genes (E). (F) Top 10 up- or downregulated gene sets between 12 h and 24 h. Dot size indicates significance (−log_10_[adjusted *p* value]). (G) GSEA showing the effects of TLC-065 on cytoprotective and antioxidative gene sets. (H) Log_2_-transformed fold changes of core genes in different DNA repair pathways. *p <0.05; **p <0.01; ***p <0.001. two-sided Student’s *t* test (A). FDR-corrected *p* values (q values) (G). BER, base excision repair; FA, fanconi anaemia; HDR, homology-dependent recombination; MMR, mismatch repair; NHEJ, non-homologous end-joining; NER, nucleotide excision repair; PARP, poly (ADP-ribose) polymerase. Resp., response; Expr., expression; Metab., metabolism; Regul., regulation.

The total cellular NAD^+^ concentration increased by ~1.5-fold within 6 h of TLC-065 treatment and the effect lasted over 24 h (Fig. 2A). Bulk RNA-seq was then performed in mPHs exposed to TLC-065 for 6 h, 12 h, or 24 h. Principal component analysis delineated groups by duration, with distinct segregation at each time point by TLC-065 (Fig. 2B). Our analysis prioritized the treatment effects by contrasting TLC-065 groups with DMSO and focused on identifying consistent changes across all timepoints (Fig. 2C-F).

TLC-065 induced 444 transcripts at all timepoints, enriched in processes related to transcription (Fig. 2C). Further, 538 genes were upregulated exclusively at 12 h and 24 h, predominantly associated with cellular stress responses (Fig. 2F). Complementing these findings, gene set-enrichment analysis showed a consistent upregulation of antioxidative pathways across all timepoints and an early enhancement of cytoprotective processes (Fig. 2G).

Four hundred and thirty-three transcripts were downregulated at three timepoints (Fig. 2E), while a greater number (818) were downregulated only at 12 h and 24 h (Fig. 2E). The universally downregulated genes were enriched in collapsed replication fork processing, DNA damage response, and DNA repair (Fig. 2C). DNA damage activates repair mechanisms, primarily involving base excision repair, mismatch repair, nucleotide excision repair, Fanconi anemia, homology-dependent-recombination, and non-homologous end-joining, among others.^22^ Core genes of these DNA repair processes were downregulated by TLC-065 in mPHs across all timepoints (Fig. 2H). Interestingly, inhibiting ACMSD downregulated the expression of several PARPs, especially PARP1, which are notable NAD^+^-consuming enzymes with roles in DNA damage repair^17,23^ (Fig. 2H).

**Fig. 3.**
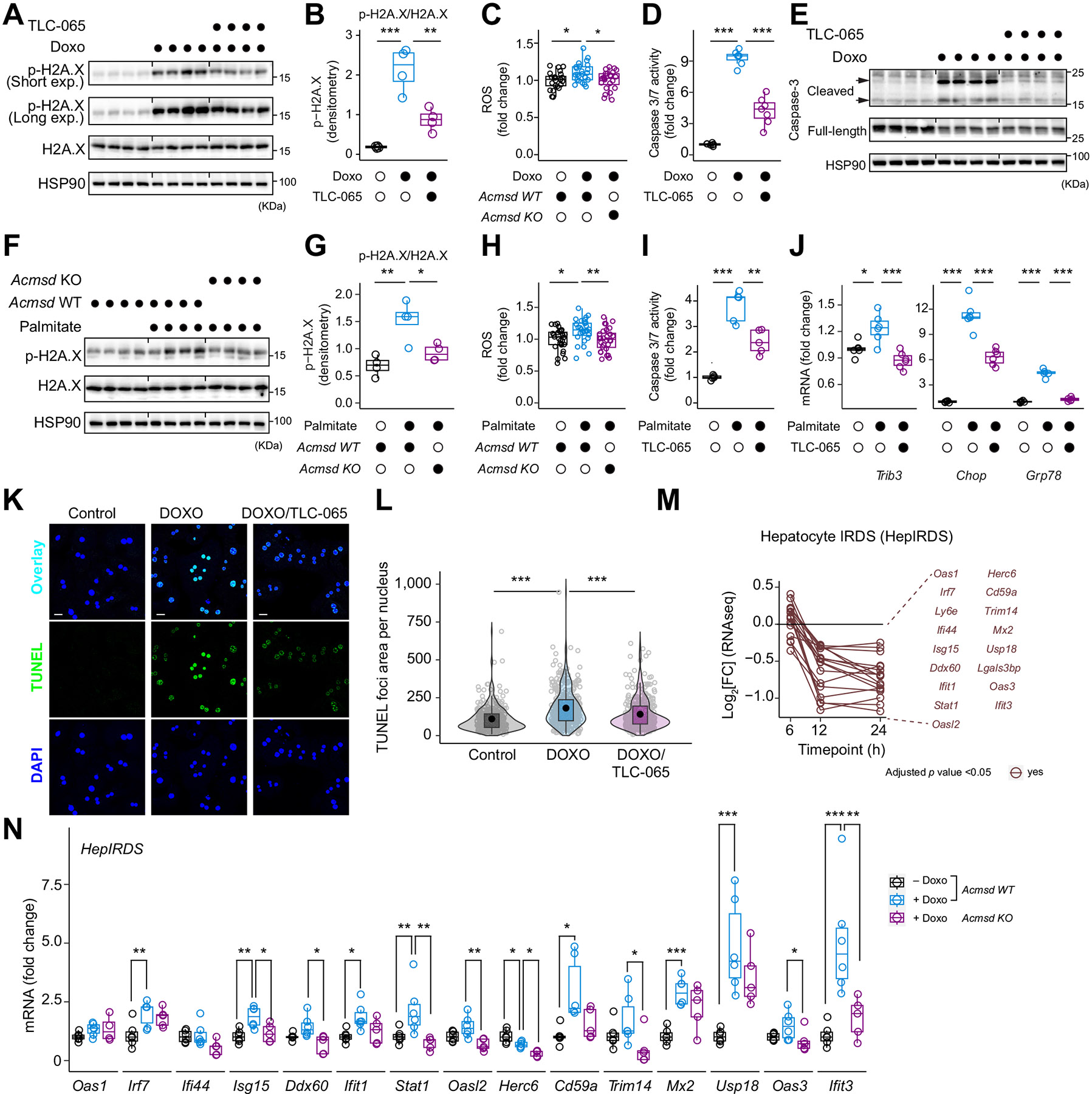
ACMSD inhibition confers genome protection in hepatocytes. (A-B) Western blot (A) and band densitometry (B) of p-H2A.X at Ser139 in mPHs (n = 4). (C) Fold changes of ROS in *Acmsd* WT or KO mPHs (n = 24). (D) Fold changes of caspase-3/7 activity in mPHs (n = 7). (E) Western blot of cleaved caspase-3 and procaspase-3 (full-length) in mPHs. For A-B, D-E, 1 μM doxorubicin ± 5 μM TLC-065 for 24h. (F–H) Western blot (F), band densitometry (G) of p-H2A.X at Ser139 (n = 4) and fold changes of ROS (n = 28) (H) in *Acmsd* WT or KO mPHs exposed to 0.75 mM palmitate. (I-J) Fold changes of caspase-3/7 activity (n = 5) (I) and mRNA of ER stress markers (n = 6) (J) in mPHs exposed to 0.75 mM palmitate ± 10 μM TLC-065 (n = 6). (K-L) TUNEL staining and foci quantification in nuclei of mPHs treated with 1 μM doxorubicin ± 2.5 μM TLC-065 for 24h (n = 290, control; n = 368, doxo; n = 329, doxo/TLC-065). Scale bar, 20 μm. (M) Log_2_ transformed FC of HepIRDS expression in mPHs after 0.5 μM TLC-065 treatment (n = 4). (N) HepIRDS expression in *Acmsd* KO or WT mPHs exposed to 0.5 μM doxorubicin (n = 5-6). *p <0.05; **p <0.01; ***p <0.001; One-way ANOVA and Tukey’s multiple comparison test (B-D; G-J; L, N). Exp., exposure; Doxo., doxorubicin; WT, wild-type; KO, knockout; HepIRDS, hepatocyte interferon-related DNA damage resistance signature.

To determine if ACMSD inhibition attenuates DNA damage response, mPHs were exposed to doxorubicin, which causes DNA double-strand breaks (DSBs) by destabilizing DNA through intercalation. Doxorubicin increased phosphorylated H2A.X levels (Ser139; p-H2A.X), a marker of DSBs, cellular reactive oxygen species production (oxidative stress), and caspase 3/7 activity (apoptosis) (Fig. 3A-D). ACMSD inhibition by TLC-065 or genetic *Acmsd* deletion attenuated these markers in doxorubicin-treated mPHs (Fig. 3A-D). Consistently, the accumulation of cleaved caspase-3, the marker of apoptosis, but not pro-caspase 3, was diminished by TLC-065 in mPHs exposed to doxorubicin (Fig. 3E and Fig. S2A). mPHs were also exposed to palmitate to model lipotoxicity-induced DNA damage, oxidative stress, apoptosis and endoplasmic reticulum stress (Fig. 3F-J). ACMSD inhibition or genetic *Acmsd* KO attenuated palmitate-induced lipotoxicity (Fig. 3F-J). Furthermore, doxorubicin-treated mPHs had significantly higher TUNEL+ foci in nuclei, indicating DNA breaks, and this was reduced by TLC-065, demonstrating the genome protection of ACMSD inhibition in hepatocytes (Fig. 3K-L).

Genes exclusively downregulated by TLC-065 treated for 12 h or 24 h were related to innate immunity, including interferon signaling, defense response to virus and IFN-stimulated genes (ISGs) (Fig. 2F and Fig. S2B). A subset of ISGs, known as the IFN-related DNA damage resistance signature (IRDS), are induced by genomic stress.^24^ Within our RNA-seq datasets, 17 out of 37 identified IRDS genes exhibited sustained down-regulation by TLC-065 in mPHs (Fig. S2C-E). We designated these 17 IRDS genes as "Hepatocyte IRDS (HepIRDS)" (Fig. 3M) that showcase the interplay between innate immunity and DNA damage in hepatocytes and the liver. Doxorubicin upregulated most HepIRDS genes in mPHs, affirming the activation of the innate immune response in hepatocytes upon genomic stress, which was attenuated by TLC-065 (Fig. 3N).

### Liver transcriptomic signatures of DNA damage correlate with MASLD/MASH severity

**Fig. 4.**
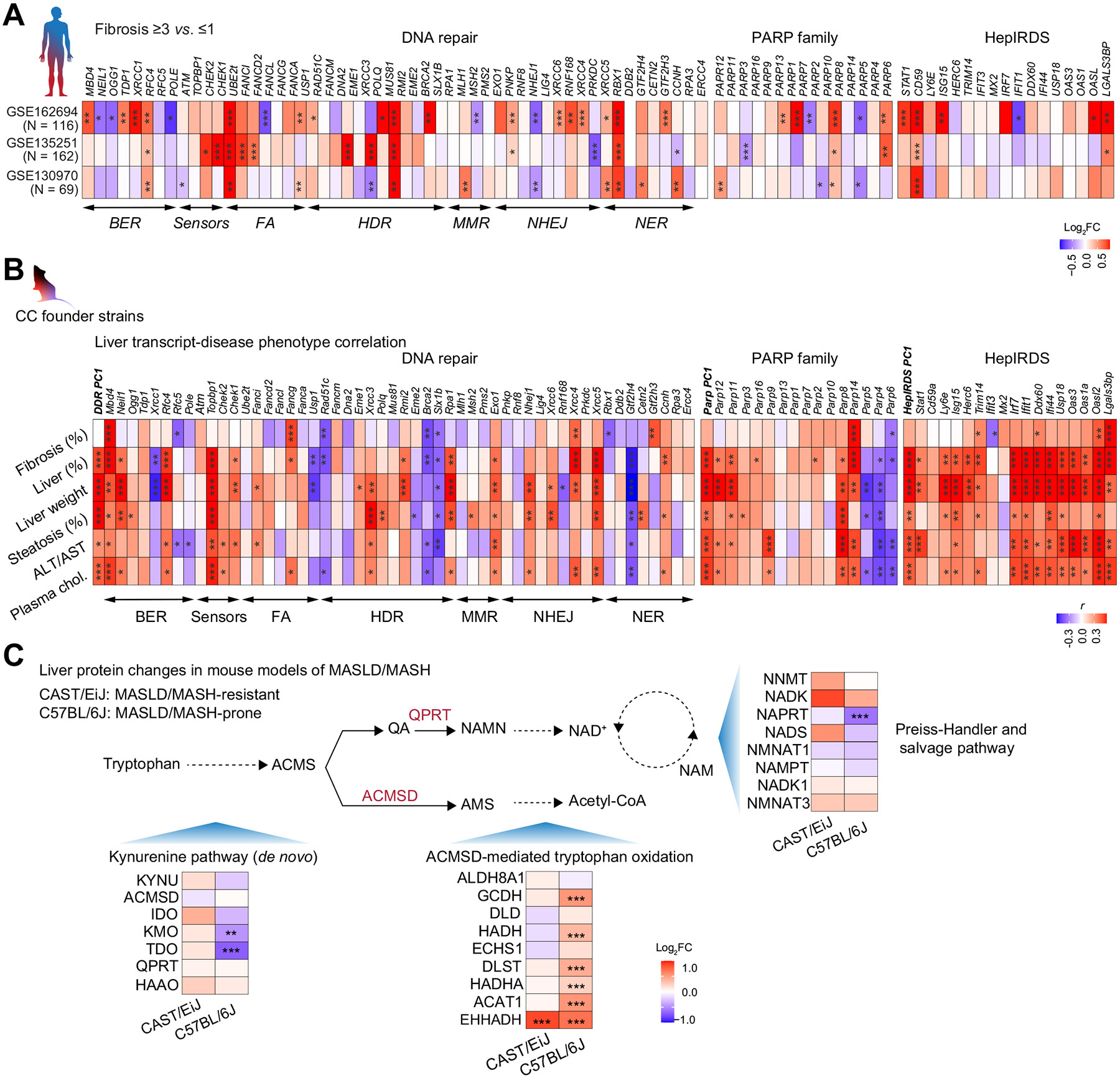
Liver DNA damage expression correlates with MASLD/MASH severity. (A) Log_2_-transformed FC of DNA repair, PARPs, and HepIRDS signature in human MASLD/MASH datasets, comparing subjects with fibrosis ≥3 *vs*. ≤1. (B) Gene and phenotype correlations in the CC founder strains. The principal component 1 (PC1) of the DNA repair (DDR), PARPs, and HepIRDS were obtained from PCA of normalized liver expression data of the CC founder strains (see Fig. S3). Liver (%), percentage of body weight. (C) Log_2_-transformed FC of liver proteins in NAD+ synthesis-related pathways in CAST/EiJ and C57BL/6J mice fed a WD diet, compared to their CD diet-fed controls. *p <0.05; **p <0.01; ***p <0.001; Benjamini–Hochberg adjusted *p* values (A-C). FC, fold change; CC founder strains, collaborative cross founder strains. Chol., cholesterol; ALT, alanine aminotransferase; AST, aspartate aminotransferase.

To understand the implications of DNA damage and the associated innate immune response in MASLD/MASH, we analyzed three human cohorts of 116, 162, and 69 individuals with comprehensive liver transcriptome and histological evaluations.^25-27^ We focused on DNA repair, the PARPs, and HepIRDS suppressed by ACMSD inhibition in mPHs (Figs 2H and 3M). Differentially expressed gene (DEG) analysis of liver transcriptome datasets comparing individuals with a fibrosis stage ≥3 to those with a fibrosis stage ≤1 showed significant upregulation of various genes within these pathways across three human MASLD/MASH cohorts (Fig. 4A).

We confirmed the association of DNA damage and innate immunity with MASLD/MASH using liver transcriptome datasets from seven genetically diverse collaborative cross founder strains exhibiting a spectrum of MASLD/MASH clinical phenotypes when fed a WD and housed at TN^28^. The first principal component (PC1) of HepIRDS, accounting for a major portion of the variance in HepIRDS gene expression (65%), along with most genes in the HepIRDS, displayed a significant correlation with markers of liver health, including ALT/aspartate aminotransferase ratio, liver percentage of body weight, liver weight, liver steatosis, and plasma cholesterol levels (Fig. 4B and Fig. S3A). Similarly, the PC1 of the DNA repair signature and PARP family correlated with a comparable array of liver health markers (Fig. 4B and Fig. S3B,C).

Notably, the MASLD/MASH-prone WD-fed C57BL/6J mouse strain exhibited impaired *de novo* NAD^+^ synthesis pathway, marked by reduced protein levels of tryptophan-2,3-dioxygenase and kynurenine 3-monooxygenase, pivotal for initial tryptophan degradation, and elevated levels of proteins downstream of ACMSD, while maintaining stable ACMSD protein levels (Fig. 4C). This change may reduce tryptophan breakdown and favor oxidation over NAD+ synthesis. In contrast, the MASLD/MASH-resistant CAST/EiJ strain showed no alteration in the NAD+ synthesis pathway following WD feeding (Fig. 4C). Thus, inhibiting ACMSD, which impedes tryptophan oxidation, may promote *de novo* NAD^+^ synthesis in MASLD/MASH, thereby mitigating disease phenotypes.

### Therapeutic intervention with a ACMSD inhibitor prevents MASLD/MASH progression

**Fig. 5.**
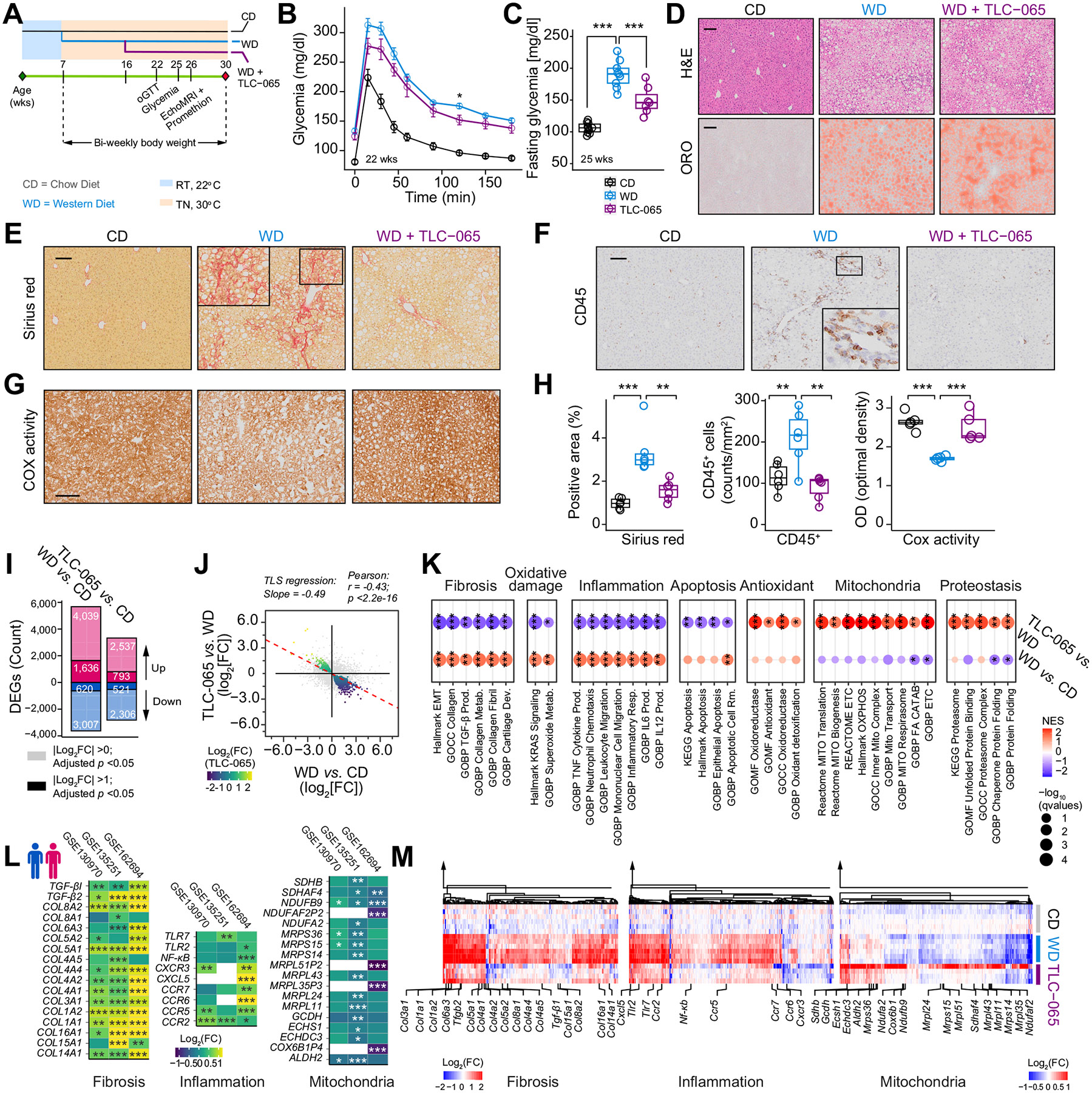
TLC-065 reverses clinical phenotypes and molecular dysregulations in MASLD/MASH. (A) Schematic of the experimental pipeline. (B) Blood glucose levels during oGTT in 22 weeks. (C) Fasting glycemia at 25 weeks (CD n = 10, WD n = 9, TLC-065 n = 7, B–C). (D) H&E of formalin-fixed liver sections and Oil red O (ORO) staining of liver cryosections (n = 6; magnification: 100 μm). (E-H) Sirius red (E), CD45 (F), and cytochrome c oxidase (COX) activity staining (G) of formalin-fixed liver sections (magnification: 100 μm). (H) Sirius red^+^ area, counts of CD45^+^ cells and optical density of COX activity staining (n = 6). (I) DEGs in WD/TN mice ± TLC-065 compared to the CD group (CD n = 6, WD n = 6, TLC-065 n = 4). (J) Comparison of the log2-transformed FC of genes under WD/TN (WD *vs*. CD) and TLC-065 (TLC-065 *vs*. WD). The dashed line shows the TLS regression line. Genes showing significant opposite changes (Benjamini–Hochberg adjusted *p* <0.05) in the comparison are color-coded by the log_2_ FC under TLC-065. (K) GSEA for gene sets affected by WD/TN (WD *vs*. CD) and reversed by TLC-065 (TLC-065 *vs*. WD). (L) Log_2_-transformed FC of fibrotic, inflammatory, and mitochondrial genes in human MASLD/MASH datasets (fibrosis ≥3 *vs*. fibrosis ≤1). (M) Log_2_-transformed FC of core genes that contribute to the enriched gene sets listed in (K). Genes presented in L are labelled in M. Error bar: mean ± SEM (B). *p <0.05; **p <0.01; ***p <0.001; One-way ANOVA and Tukey’s multiple comparisons test (C, H). FDR-corrected *p* values (q values) (K); Benjamini–Hochberg adjusted *p* values (I, L). oGTT, oral glucose tolerance test; CD, a chow (control) diet; WD, a western-style diet; ORO, Oil Red O; DEGs, differentially expressed genes; FC, fold change; TLS regression, total least squares regression; NES, normalized enrichment score; Prod., production; Metab., metabolism; Dev., development; Resp., response; Rm., removal; MITO/Mito, mitochondria; ETC, electron transport chain; OXPHOS, oxidative phosphorylation; FA, fatty acid; CATAB, catabolism.

We next evaluated the impact of TLC-065 on MASLD/MASH progression in a dietary mouse model of MASLD/MASH. C57BL/6J male mice were housed at TN and fed a WD diet (WD/TN) at 7 weeks of age. Housing mice at TN (~30 °C) is more effective at inducing liver damage than housing at ambient temperature (~22 °C).^29^ TLC-065 (25 mg/kg/day, admixed in WD) was given in a therapeutic regimen after 9 weeks of WD/TN (Fig. 5A). TLC-065 is orally bioavailable and we measured plasma concentrations of ~389.7 ng/ml at the end of the study (Fig. S4A). TLC-065-treated animals appeared to have increased food intake in the study (Fig. S4B). While body weight was not different, lower adiposity was observed in TLC-065-treated mice compared with control mice (Fig. S4C-F). Energy expenditure tended to increase in TLC-065-treated mice, particularly in the dark phase, while locomotor activity remained unaffected (Fig. S4G-J). WD/TN compromised glucose tolerance and fasting glycemia; TLC-065 partially restored fasting glycemia without affecting glucose tolerance (Fig. 5B,C and Fig. S4K).

Hepatic steatosis was evident at the end of the study, characterized by substantial hepatic lipid deposition, a pale white liver coloration, and an over two-fold increase in liver weight (Fig. 5D and Fig. S4L-O); these steatosis-related parameters were not affected by TLC-065 (Fig. 5D and Fig. S4L-O). However, TLC-065 significantly reduced hepatic collagen content, reversed the infiltration of CD45-positive immune cells, improved cytochrome c oxidase activity in the liver (Fig. 5E-H), indicative of attenuated inflammation and fibrosis, and enhanced mitochondrial respiration. Plasma ALT levels were increased by WD/TN, but this increase was not reversed by TLC-065 (Fig. S4P). TLC-065 was well tolerated, as evidenced by the absence of an impact on kidney function, pancreatic and muscle enzymes (Fig. S4Q-S). Furthermore, TLC-065 reduced the increase of plasma cholesterol in WD/TN mice (Fig. S4T).

### ACMSD inhibition reverses MASLD/MASH-associated molecular dysregulations

We then performed bulk RNA-seq on livers from this study. Differential analysis revealed a large number of transcripts affected by WD/TN and this effect was reversed by TLC-065 (Fig. 5I-J). Pearson correlation showed a negative relationship between WD/TN-induced changes and therapeutic effects conferred by TLC-065 (r = −0.43; *p* <2.2e-16) (Fig. 5J). To estimate the magnitude of the reversal, we used total least squares regression to estimate the slope of the association and found that TLC-065 reversed nearly half of the effects of WD/TN on average, with a coefficient of −0.49 (Fig. 5J). Concordant with the histological reversal of fibrosis and inflammation, TLC-065 countered the upregulation of fibrosis and inflammation-related gene signatures, such as collagen fibril production and mononuclear cell infiltration, and increased transcripts associated with mitochondrial respiration and biogenesis (Fig. 5K). Furthermore, TLC-065 reversed oxidative damage and apoptosis gene signatures induced by WD/TN, while enhancing the expression of genes enriched in cytoprotection, antioxidant responses, and proteostasis (Fig. 5K and Fig. S5A).

To determine whether genes dysregulated in WD/TN mice were similarly affected in human MASLD/MASH, we profiled DEGs in the previously mentioned human MASLD cohorts^25-27^ (Fig. 5L). The transcripts most changed in human MASLD/MASH were also among the core contributors to fibrotic progression, inflammatory activation, and suppression of mitochondrial function in WD/TN mice (Fig. 5L,M). Expression of the transcripts involved in these processes was reversed by TLC-065 in our mouse studies (Fig. 5M).

### ACMSD inhibition counters cell population shifts and DNA damage in MASLD/MASH

**Fig. 6.**
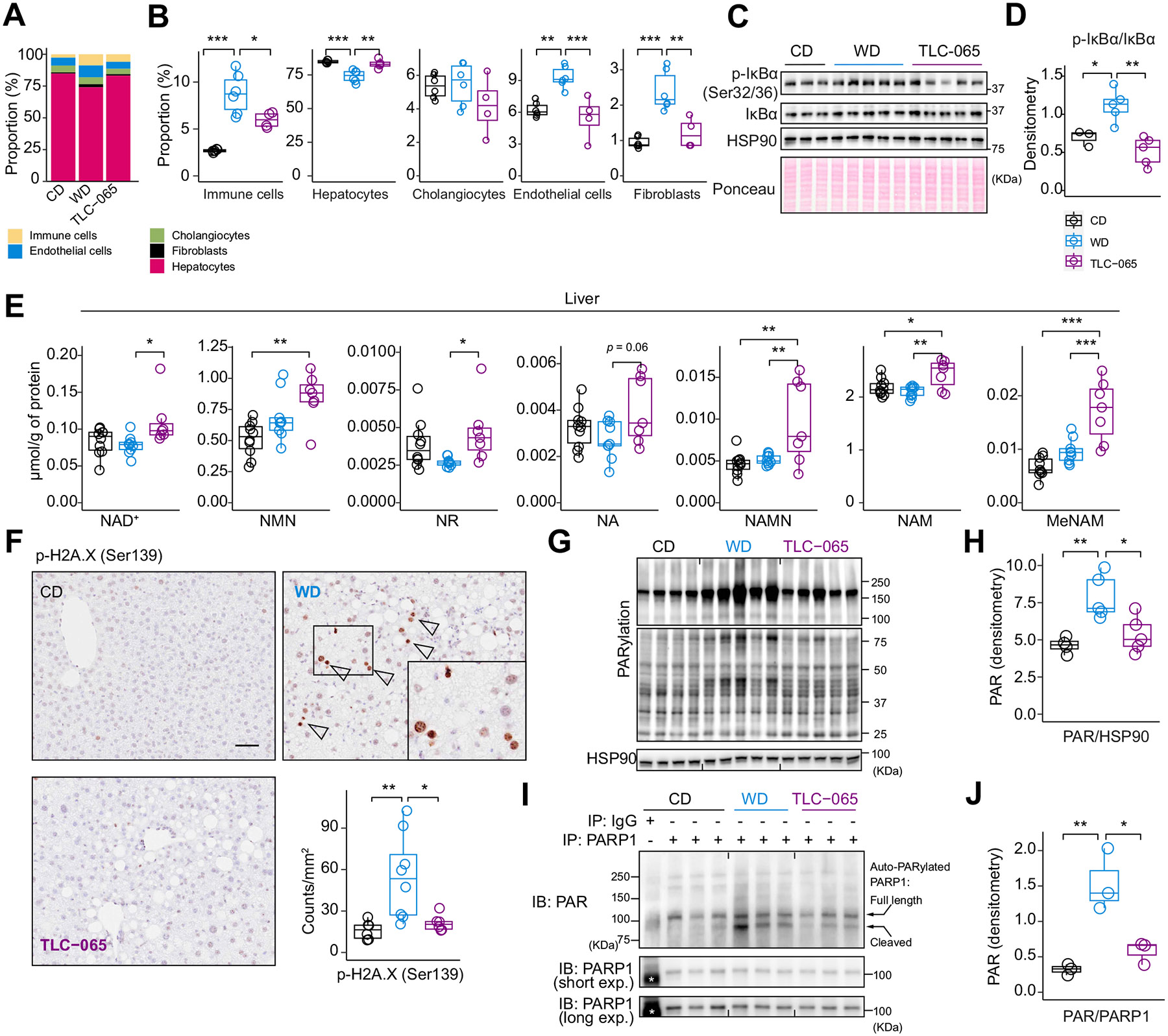
TLC-065 counters liver cell shifts and liver DNA damage in WD/TN mice. (A-B) Single-cell deconvolution indicating the estimated percentage of liver cell types shown in stacked bar graph (A) and boxplot (B). (C-D) Western blot of phosphorylated IκBα at Ser32/36 and total IκBα in liver lysates (CD n = 3, WD n = 5, TLC-065 n = 5) (C) and band densitometry of p-IκBα normalized to total IκBα (D). (E) Abundance of liver NAD+ metabolome normalized to protein (CD n = 10, WD n = 9, TLC-065 n = 7). (F) p-H2A.X (ser139) staining and quantification of formalin-fixed liver sections (magnification: 50 μm) (n = 6, CD; n = 8, WD; n = 6 TLC-065). Arrows indicate p-H2A.X^+^ nuclei. (G-H) Western blot of PARylation in liver lysates (G) and band densitometry of PAR normalized to HSP90 (H) (I-J) Immunoblotting of PAR and PARP1 on immunoprecipitated PARP1 (I) and band densitometry of auto-PARylated PARP1 normalized to total immunoprecipitated PARP1 (n = 3, CD; n = 3, WD; n = 3 TLC-065) (J). Stars in immunoblots indicate non-specific bands. In the image, the full-length PARP1 (116 KDa) and the cleaved PARP1 fragment (89 KDa) are labelled. *p <0.05; **p <0.01; ***p <0.001. One-way ANOVA and Tukey’s multiple comparisons test (B, D-F, H-J). NAD^+^, nicotinamide adenine dinucleotide; NMN, nicotinamide mononucleotide; NR, nicotinamide riboside; NA, nicotinic acid; NAMN, nicotinic acid mononucleotide; NAM, nicotinamide; MeNAM, methyl-NAM; PAR, poly-ADP ribose; Exp., exposure.

To assess the heterogeneity and shifts in specific hepatic cell populations in MASLD/MASH, we performed single-cell deconvolution on the liver bulk RNA-seq data. This analysis showed that livers of WD/TN mice have a higher proportion of immune cells, endothelial cells and fibroblasts, concomitant with a decreased proportion of hepatocytes (Fig. 6A,B and Fig. S5B). These pathogenic shifts in liver cell populations were reversed by TLC-065 (Fig. 6A,B and Fig. S5B). In line with the decrease of immune cells, TLC-065 reversed the increased phosphorylation of IκBa at Ser32/36 in WD/TN mice (Fig. 6C,D), suggesting a suppression of NF-κB activation after TLC-065 treatment.

Deep-targeted metabolomics was conducted on liver and quadriceps to determine the impact of TLC-065 on NAD^+^ metabolome. In the liver of WD/TN mice, TLC-065 elevated the levels of NAD^+^ and the associated salvage metabolites, including NR, NAMN and NAM without impacting levels of metabolites upstream of ACMSD, *e.g.* KYN and KYN acid (Fig. 6E and Fig. S5E). In contrast, this effect was absent in quadriceps (Fig. S5C and D), consistent with specific ACMSD expression in the liver (Fig. S5F and G).

WD/TN upregulated DNA repair pathways and several PARP members, especially *Parp*1 and *Parp*2 known for their activation in response to genomic stress^23^ (Fig. S6A). TLC-065 partially reversed this activated DNA repair profile with notable effects on the expression of *Parp1, Parp4, Parp8*, and *Atm* (Fig. S6A). The HepIRDS was also activated by WD/TN and partially mitigated by TLC-065 (Fig. S6B). TLC-065 also suppressed WD/TN-induced innate immune activation which could be attributed to the reduced expression of ISGs and NF-κB target genes (Fig. S6C-D).

WD/TN livers had increased numbers of p-H2A.X (Ser139)-positive cells (Fig. 6F) relative to CD-fed mice, indicating activation of DNA damage. TLC-065 reversed this phenotype with normalization of the number of p-H2A.X-positive cells to levels comparable to those observed in CD-fed mouse livers (Fig. 6F), consistent with the protective effects of TLC-065 observed in mPHs (Fig. 3). We next assessed the effect of TLC-065 on global poly-ADP ribosylation (PARylation), indicative of PARP1 and PARP2 activation by DNA damage, and the auto-PARylation of PARP1. WD/TN increased the hepatic levels of global PARylation and auto-PARylated PARP1, particularly in its cleaved form, compared to CD (Fig. 6G-J). TLC-065 abrogated these increases, reducing both global PARylation and auto-PARylated PARP1 levels (Fig. 6G-J), in line with its genome-protective effects.

### The effects of ACMSD inhibition are recapitulated in sHLO models

**Fig. 7.**
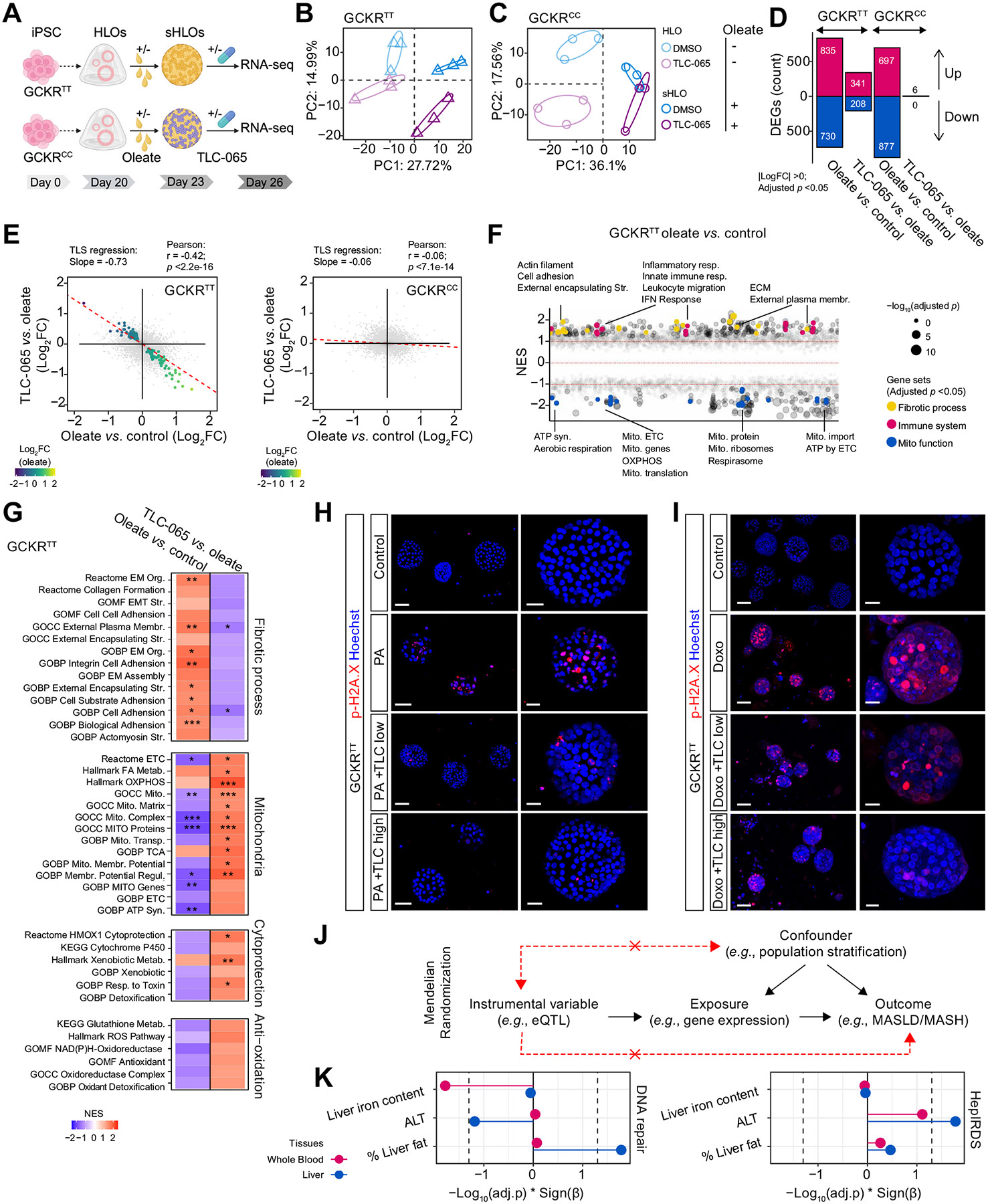
Effects of ACMSD inhibition are recapitulated in HLO steatohepatitis models. (A) Schematic of the experimental pipeline. Created with Biorender.com. (B–C) PCA of normalized gene expression of GCKR^TT^ (B) and GCKR^CC^ (C) sHLOs ± 10 μM TLC-065 (n = 3). (D) DEGs induced by oleate or TLC-065 in GCKR^TT^ or GCKR^CC^ HLOs. (E) Comparison of the log2-transformed FC of genes under oleate (oleate *vs*. control) and TLC-065’s effects (TLC-065 *vs*. oleate) in GCKR^TT^ or GCKR^CC^ sHLOs. The dashed line shows the TLS regression line. Genes showing significant opposite changes (Benjamini–Hochberg adjusted *p* <0.05) in the comparison are color-coded by the log2 FC under oleate. (F) Manhattan plot showing GSEA for gene sets affected by oleate in GCKR^TT^ sHLOs. Significant gene sets are colored as indicated for different processes. (G) GSEA of gene sets induced by oleate (oleate *vs*. control) and reversed by TLC-065 (TLC-065 *vs*. oleate) in GCKR^TT^ sHLOs. (H–I) p-H2A.X (Ser139) immunostaining of GCKR^TT^ HLOs exposed to 300 μM palmitate (PA) for 48 h (H) or to 5 μM doxorubicin for 24 h (I) ± 1 μM (low) or 10 μM (high) TLC-065. p-H2A.X (ser139) staining (red) and Hoechst33342 (blue). Low magnification: 50 μm for PA, PA + TLC and 70 μm for other groups. High magnification: 30 μm for control, PA, PA + TLC and 20 μm for other groups. (J) Schematic of Mendelian randomization analysis. (K) The effects of liver and whole blood DNA repair and HepIRDS gene expression on liver iron content, plasma ALT and liver fat percentage. *p <0.05; **p <0.01; ***p <0.001; Benjamini–Hochberg adjusted *p* values (D, K). FDR-corrected *p* values (F, G). iPSC, induced pluripotent stem cell; HLOs, human liver organoids; sHLOs, steatohepatitic HLOs; DEG, differentially expressed genes; Mito., mitochondria; Str., structure; Resp., response; IFN, interferon; Membr., membrane; ECM/EM, extracellular matrix; Syn., synthesis; Org., organization; Metab., metabolism; Transp., transport; Regul., regulation.

To ascertain the translation of mechanisms associated with ACMSD inhibition from mice to humans, we employed induced pluripotent stem cell-derived HLOs harboring the glucokinase regulatory protein (GCKR) rs1260326:C>T variant.^30^ To model steatohepatitis in HLOs (sHLOs), HLOs were treated with oleate for 3 days and this was followed by another 3-day TLC-065 treatment (Fig. 7A). GCKR^TT^ sHLOs exhibited greater steatosis phenotypes, as evidenced by more lipid accumulation than GCKR^CC^ sHLOs, and this was not affected by TLC-065 (Fig. S7A and B), consistent with the lack of an effect of TLC-065 on liver steatosis in WD/TN mice (Fig. 5D).

We then performed bulk RNA-seq on TLC-065-treated sHLOs (Fig. 7A). Principal component analysis of GCKR^TT^ HLOs revealed separation of groups into four quadrants based on oleate and TLC-065, explaining 27.72% and 14.99% variance, respectively (Fig. 7B). In contrast, the GCKR^CC^ HLOs segregated into three quadrants, with the sHLO groups converging within a shared cluster, irrespective of TLC-065 treatment (Fig. 7C). Differential analysis showed that while oleate induced a comparable number of DEGs in GCKR^TT^ and GCKR^CC^ sHLOs, the effects of TLC-065 were specific only to the GCKR^TT^ sHLOs (Fig. 7D). Further, in GCKR^TT^ sHLOs, regression and correlation analysis revealed a pronounced negative relationship between oleate-induced changes and the effects of TLC-065 (r = −0.42*p* <2.2e-16) and more than 70% of the effects of oleate were reversed by TLC-065, with a coefficient of −0.73 (Fig. 7E). Conversely, in GCKR^CC^ sHLOs, no such correlation was observed (Fig. 7E). These results show that the effects of TLC-065 are restricted to GCKR^TT^ sHLOs, which exhibit more severe steatosis phenotypes (Fig. S7A and B).

To further elucidate the mechanisms driving the distinct outcomes associated with TLC-065 treatment, we compared the dysregulated processes in GCKR^TT^ and GCKR^CC^ sHLOs. Upon oleate treatment, HLOs exhibited steatohepatitis-like phenotypes including upregulation of fibrosis, immune and inflammatory responses, independent of GCKR genotypes (Fig. 7F and Fig. S7C). However, gene signatures associated with various mitochondrial respiratory functions were downregulated only in GCKR^TT^ sHLOs (Fig. 7F and Fig. S7C), consistent with previous reports that the impairment of mitochondrial oxidative phosphorylation was associated with the TT variant.^30^ Notably, TLC-065 attenuated the suppression of mitochondrial respiratory processes, mitigated the induction of fibrotic pathways, and enhanced cytoprotective and antioxidative mechanisms in GCKR^TT^ sHLOs (Fig. 7G).

We next verified whether inhibiting ACMSD suppresses genotoxic stress in sHLOs induced by oleate. Gene expression profiles of DNA repair, PARPs and HepIRDS remained unaltered in either GCKR^TT^ or GCKR^CC^ sHLOs (Fig. S7D). However, in the presence of palmitate, a saturated fatty acid that causes more pronounced lipotoxicity than oleate,^9^ GCKR^TT^ HLOs demonstrated an accumulation of DNA lesions, detected by the formation of p-H2A.X (S139) and p-53BP1 (Ser1778) foci in nuclei, two canonical markers of DSBs (Fig. 7H and Fig. S7E,G,I). TLC-065 attenuated palmitate-induced p-H2A.X and p-53BP1 accumulation (Fig. 7H; Fig. S7E, G, I). To examine the effects of ACMSD inhibition against direct genomic stress, GCKR^TT^ HLOs were exposed to doxorubicin. Doxorubicin led to significant formation of p-H2A.X and p-53BP1 foci, and this DNA-damaging effect was alleviated by TLC-065 (Fig. 7I and Fig. S7F,H,I).

To determine if DNA damage also accounts for differences in liver disease susceptibility in humans, we performed Mendelian randomization to investigate the relationship between gene expression of DNA damage-related signatures, including DNA repair and the HepIRDS, and liver disease incidence (Fig. 7J). The results of this analysis revealed that higher expression of DNA repair genes in the liver increased the percentage of liver fat (*p* = 2.6*10^−4^, q = 0.017), while higher expression of the HepIRDS genes in liver or in whole blood increased the plasma levels of ALT (*p* = 5.5*10^−4^, q = 0.017 for liver, *p* = 4.3*10^−3^, q = 0.078 for whole blood) (Fig. 7K). Thus, our genetic analysis provides further evidence on the contribution of DNA damage to liver disease susceptibility in humans.

## Discussion

Our data demonstrate that inhibiting ACMSD using TLC-065, a selective and potent ACMSD inhibitor, not only augments the NAD^+^ metabolome, enhances mitochondrial respiration, and improves antioxidative stress in hepatocytes,^18^ but also attenuates genomic stress and innate immune activation, phenomena corroborated *ex vivo, in vivo* and in HLOs. Our approach of combining a WD with TN housing in mice recapitulates various pathological aspects of human MASLD/MASH, including liver steatosis, hepatic inflammation and fibrosis, and mitochondrial impairment (Fig. 5 and Fig. S4). While the NAD^+^ *de novo* synthesis pathway was impaired in WD/TN mice (Fig. 4C), this did not result in hepatic NAD^+^ decrease (Fig. 6E). However, ACMSD inhibition with TLC-065 increased hepatic NAD^+^ levels (Fig. 6E). These results suggest that raising hepatic NAD^+^ above baseline is key to the therapeutic effects of ACMSD inhibition on MASLD/MASH. In addition to increasing NAD^+^, inhibiting ACMSD likely prevents acetyl-CoA production from tryptophan. The two effects may synergistically contribute to the metabolic and genomic benefits observed in mPHs, mouse liver, and HLOs.

A distinguishing benefit of ACMSD inhibition is its genomic-protective effects observed *ex vivo*, in mouse livers and HLOs. This protective mechanism is potentially mediated by augmented NAD^+^ bioavailability to nuclear PARPs, facilitating enhanced DNA repair capacity upon genotoxic stress. This, in turn, mitigates the accumulation of DNA lesion and averts hyperactivation of PARPs, which otherwise leads to further NAD^+^ depletion, precipitating cellular demise through various apoptotic pathways.^31^ Here, we demonstrate that inhibiting ACMSD elevates liver NAD^+^ content and diminishes DNA damage levels, thereby normalizing PARP1/2-induced PARylation and protecting livers of WD/TN mice.

In the MASLD/MASH livers, accumulation of DNA damage resulting from genome instability,^9,32^ oxidative stress^33^ and replication stress^8^ is evident. Recent studies, using advanced technologies capable of detecting hepatic mutations at a single-cell level or within small liver clones have uncovered an elevated burden of somatic mutations in human MASLD/MASH, further corroborating genomic instability under these disease conditions.^34-36^ Our transcriptomic analysis in mice and humans showed a positive correlation between the expression of DNA damage-related signatures and the liver phenotypes of MASLD/MASH. Mendelian randomization analysis substantiated the significant effects of increased expression of DNA damage-related signatures on liver fat percentage and plasma ALT levels in humans, thereby highlighting the causal role of DNA damage in the pathogenesis of MASLD/MASH.

In sHLOs, TLC-065 exhibits distinct treatment responses based upon the single-nucleotide polymorphisms at the *GCKR* locus, specifically rs1260326:C>T. Compared to GCKR^CC^, GCKR^TT^ HLOs show enhanced steatosis phenotypes particularly upon fat loading with oleate^30^ (Fig. S7A and B). This genetic susceptibility to steatosis arises from the impact of the TT variant on protein activity. GCKR is a hepatocyte-specific gene and GCKR-rs1260326:C>T is a functional risk variant which encodes a Pro446Leu missense mutation that inhibits the activity of the glucokinase regulatory protein.^37^ This inhibition leads to higher glucokinase activity, constant activation of glycolysis, and excess production of acetyl-CoA, a key substrate for *de novo* lipogenesis, promoting fat accumulation.^30^ The GCKR^TT^ has been postulated to be the causal variant that underlies the increased risk of MASLD/MASH associated with GCKR variants.^37^ The stronger effects of TLC-065 in the GCKR^TT^ context are likely related to the increased disease severity upon fat loading conferred by the TT variation (Fig. S7A and B). Particularly, mitochondrial respiration, which benefits from ACMSD inhibition (Fig. 1), appears to be more impaired in GCKR^TT^ HLOs upon oleate treatment (Fig. 7F and Fig. S7C). This underscores mitochondria-centric mechanisms that likely underlie the therapeutic action of TLC-065.

In initial clinical trials, administration of NAD^+^ precursors, such as NR and NMN, to people with obesity or insulin resistance, conditions associated with MASLD/MASH, demonstrated only minor effects.^38-40^ It hence remains uncertain whether oral administration of NAD^+^ precursors in humans elevates hepatic NAD^+^ to therapeutically relevant concentrations. ACMSD inhibition in contrast can achieve tissue-specific boosting effects on NAD^+^ content, owing to the restricted expression of this enzyme primarily in the liver and kidney. Our study showed that increasing NAD^+^ by ACMSD inhibition exhibited hepatocyte-specific and liver-autonomous effects on mitochondrial energetics, antioxidative responses, and genomic stability and demonstrated the therapeutic potential to reverse the MASLD/MASH phenotypes in both mice and human liver organoids. Our data advocate that targeting ACMSD to promote *de novo* NAD^+^ production in the liver holds promise for further development in humans as an innovative approach to preserve hepatic function in MASLD/MASH.

## Supplementary Material

Supplementary Information

## Data Availability

Raw RNA-seq data generated in this study are available from Gene Expression Omnibus under the accession numbers GSE253217 and GSE253380. Other data associated with this paper are available upon request to the corresponding author. Reagents, antibodies and resources are listed in the material and methods section, supplementary materials and methods and in the CTAT table.

## Highlights

- ACMSD inhibition enhances NAD^+^ *de novo* synthesis and enables genome stability in hepatocytes.
- ACMSD inhibition increases NAD^+^ levels, reduces DNA damage and prevents MASLD/MASH progression.
- The therapeutic benefits of ACMSD inhibition are recapitulated in human liver organoid models of steatohepatitis.
- Mendelian randomization suggests causality of DNA damage in MASLD/MASH incidence.

## Impact and implications

Enhancing NAD^+^ levels has been shown to induce remarkable health benefits in mouse models of metabolic dysfunction-associated steatotic liver disease/steatohepatitis (MASLD/MASH), yet liver-specific NAD^+^ boosting strategies remain underexplored. Here, we present a novel pharmacological approach to enhance *de novo* synthesis of NAD^+^ in the liver by inhibiting μ-amino-β-carboxymuconate-ε-semialdehyde decarboxylase (ACMSD), an enzyme highly expressed in the liver. Inhibiting ACMSD increases NAD^+^ levels, enhances mitochondrial respiration, and maintains genomic stability in hepatocytes *ex vivo* and *in vivo*. These molecular benefits prevent disease progression in both mouse and human liver organoid models of steatohepatitis. Our preclinical study identifies ACMSD as a promising target for MASLD/MASH management and lays the groundwork for developing ACMSD inhibitors as a clinical treatment.

## Abbreviations

ACMSD: α-amino-β-carboxymuconate-ε-semialdehyde decarboxylase
CD: control diet
FC: fold change
HepIRDS: hepatocyte IRDS
DDR: DNA damage repair
FAO: fatty acid oxidation
HLOs: human liver organoids
IRDS: interferon-related DNA damage resistance signature
PARPs: poly (ADP-ribose) polymerases
oGTT: oral glucose tolerance test
mPH: mouse primary hepatocytes
TN: thermoneutrality
WD: western-style diet
