# Supplementary material for: ACMSD inhibition corrects fibrosis, inflammation, and DNA damage in MASLD/MASH
Source: J Hepatol. Author manuscript; Available in PMC 2025 Aug 1. (PMC11741923; doi:10.1016/j.jhep.2024.08.009)
Supplement: Supplementary Information [file NIHMS2033184-supplement-Supplementary_Information.pdf]

ACMSD inhibition corrects fibrosis, inflammation, and DNA damage in MASLD/MASH

Yasmine J. Liu, Masaki Kimura, Xiaoxu Li, Jonathan Sulc, Qi Wang, Sandra Rodríguez-López, Angelique M.L. Scantlebery, Keno Strotjohann, Hector Gallart-Ayala, Archana Vijayakumar, Robert P. Myers, Julijana Ivanisevic, Riekelt H. Houtkooper, G. Mani Subramanian, Takanori Takebe, Johan Auwerx

Table of contents

Supplementary materials and methods 2

Fig. S1 7

Fig. S2 8

Fig. S3 9

Fig. S4 10

Fig. S5 11

Fig. S6 12

Fig. S7 13

Tables S1-7……………………………………………...………..separate excel files

Table S8 14

Supplementary references 15

Supplementary materials and methods

mtDNA/nuclear DNA ratio

The mtDNA/nuclear DNA ratio was measured as previously described[1]. Briefly, total DNA was extracted with the Nucleospin Tissue Kit (#740952, Macherey Nagel). Quantitative PCR (qPCR) was performed on the Light-Cycler system (Roche Applied Science) with the following primers: 16s rRNA Fwd (5′–3′): CCGCAAGGGAAAGATGAAAGAC, Rev: TCGTTTGGTTTCGGGGTTTC; ND4 Fwd: AACGGATCCACAGCCGTA, Rev: AGTCCTCGGGCCATGATT; HK2 Fwd: GCCAGCCTCTCCTGATTTTAGTGT, Rev: GGGAACACAAAAGACCTCTTCTGG; The relative mtDNA/nuclear DNA ratio was computed by the ΔΔCt method.

ATP quantification

ATP of mPHs was quantified with ATP assay kit (#ab83355, Abcam). Briefly, about 3×10^5 cells were harvested from each well of 6-well plates. Washed with cold PBS and homogenized in 100 µL ATP assay buffer provided in the kit, followed by centrifugation at 13,000 g in cold for 5 min. Supernatant was transferred to new tubes and ATP was quantified using glycerol phosphorylation-coupled product generation that can be quantified fluorometrically with excitation and emission wavelength of 535/587 nm respectively.

Animal studies

Body composition analysis (Echo-MRI)

At 26 wks of age, body composition was evaluated using an Echo-MRI (the 3-in-1; Echo Medical Systems). This technology measured each mouse's lean and fat mass, as well as their overall body mass/weight, in about one minute per subject.

Promethion metabolic cage

At 26 wks of age, the mice were housed in the metabolic and behavioral phenotyping system (Promethion; Sable Systems) for 48h. Mice were housed in individual metabolic cage and movement, and oxygen consumption (VO2), and carbon dioxide production (VCO2) were measured every 3 min. The first 24h were considered adaptation, and the second 24 h were used for data analysis.

Oral glucose tolerance test (oGTT)

Mice were fasted overnight. In the morning of oGTT experiment, mice received a gavage of a 20% glucose solution in water (2 g glucose/kg body weight) to induce hyperglycemia. Blood glucose levels were measured from the tail vein using a glucometer before the gavage and 15, 30, 45, 60, 90, 120, 150, and 180 min after.

Plasma biochemistry

Plasma parameters were measured on 2 times diluted plasma samples using Dimension Xpand Plus (Siemens Healthcare Diagnostics AG). The biochemical tests were performed according to the manufacturer kit for each parameter: enzymatic creatinine (DF270B; Siemens Healthcare), cholesterol (DF27; Siemens Healthcare), transaminase ALAT (DF143; Siemens Healthcare), urea nitrogen (DF21; Siemens Healthcare), Amylase (Siemens Healthcare, DF17A), Creatine kinase (Siemens Healthcare, DF38).

Deep targeted or multiple pathway targeted analysis of polar metabolome in cell lysates
Cell culture sample preparation. At sampling time-point, cell cultures were rapidly rinsed with PBS (at room temperature, x2) to wash off the spent media. Cells were then extracted by the addition of 1000 µL of ice-cold MeOH:H_2O (4:1), scraped and homogenized in the Cryolys Precellys 24 sample Homogenizer (2 x 20 seconds at 10000 rpm, Bertin Technologies, Rockville, MD , US) with ceramic beads. The bead beater was air-cooled down at a flow rate of 110 L/min at 6 bars. Homogenized extracts were centrifuged for 15 min at 4000 g at 4°C (Hermle, Gosheim, Germany) and the resulting supernatant was collected and evaporated to dryness in a vacuum concentrator (LabConco, Missouri, US). Dried sample extracts were resuspended in MeOH:H_2O (4:1, v/v) according to the total protein content, measured using BCA assay (Thermo Scientific). **LC-MS/MS analysis.** Cell extracts were analyzed by high performance liquid chromatography coupled to tandem mass spectrometry (HPLC-MS/MS) in both positive and negative ionization modes using a 6495 triple quadrupole system (QqQ) interfaced with 1290 UHPLC system (Agilent Technologies). **In positive mode and to cover NAD^+ metabolome**, the chromatographic separation was carried out using the Scherzo SMC18 (3 µm, 2.0 mm x 150 mm) column (Imtakt, Portland, USA). The mobile phase was composed of A = 20 mM ammonium formate and 0.1% formic acid in H_2O and B = 20 mM ammonium formate and 0.1% formic acid (90:10, v/v) in acetonitrile. The chromatographic gradient, ESI source conditions and MRM parameters are detailed in the previously published van der Velpen et al. 2021[2]. Quality Control (QC) pooled samples (representative of the entire sample set) were analyzed periodically throughout the sample batch. **Data processing.** Raw LC-MS/MS data was processed using the Agilent Quantitative analysis software (version B.07.00, MassHunter Agilent technologies). Relative quantification of metabolites was based on EIC (Extracted Ion Chromatogram) areas for the monitored MRM transitions. Signal intensity drift correction was done using QC samples and MRM PROBS software. Peaks with poor analytical reproducibility (CV > 30%) were discarded from further statistical analysis. Metabolomics data are provided in Table S1.

Targeted quantification of NAD^+ metabolome in tissue lysates
Tissue sample preparation. Snap-frozen_liver and quadriceps samples were ground into powder using the Cryolys Precellys 24 as specified above. Each sample was then pre-weighed (~30-40 mg) into lysis tubes (soft tissue homogenizing CK 14 tubes) and stored at −80 °C prior to metabolite extraction. Metabolites were further extracted by the addition of 600-700 µL (adjusted to sample weight) of ice-cold MeOH:H_2O (4:1), homogenized in the Cryolys Precellys and centrifuged as described above. For NAD^+ metabolome quantification, 100 µL of tissue lysate aliquot was mixed with 25 µL of ISTD mixture and 125 µL of MeOH, evaporated to dryness (to pre-concentrate the extract) and reconstituted in 70 µL of H_2O for injection. The second aliquot (500 µL for liver and 350 µL for quadriceps) was first evaporated to dryness and reconstituted in 200 µL of ISTD mixture in MeOH:H_2O (4:1, v/v). The ISTD mixture contained 13C labeled AMP, ADP, ATP and NAD^+, as internal standards. Calibrators were prepared fresh, the day of the experiment, using MeOH:H_2O (4:1, v/v) purged with argon, and extracted further (through ISTD spike) in the same way as samples. The calibration range was from 0.012 to 100 µM for all metabolites except NAD^+, for which the starting concentration was 10x higher. **LC-MS/MS analyses of NAD^+ metabolome.** The NAD^+ metabolites (including the intermediates in *de novo* synthesis and salvage pathways) were quantified in the same LC-MS/MS conditions as previously[2]. Calibrators and samples were prepared in the same way, using the ISTD spike for metabolite quantification. **LC-MS/MS analyses of redox couples NAD^+/NADH, NADP+/NADPH.** Indicated redox couples were quantified in tissue lysates in

the same analytical conditions as previously described[3]. These conditions were adapted with the ISTD spike and calibrator preparation (as described above) to allow for metabolite quantification. **Data processing.** Raw LC-MS/MS data was processed using the Agilent Quantitative analysis software (version B.07.00, MassHunter Agilent technologies). Peak areas were based on EIC (Extracted Ion Chromatogram) areas for the monitored MRM transitions and translated into concentrations using calibration curves and ISTD spike (i.e., response factor). The concentrations were reported to total protein content (determined using BCA assay). Metabolomics data are provided in Table S5.

RNA-Seq and mapping

For RNA-seq analyses, SOAPnuke software[4] was applied to remove adapters and low-quality reads and the quality check of these samples was performed by FastQC (https://www.bioinformatics.babraham.ac.uk/projects/fastqc/). Clean reads were mapped to the mouse genome (Mus musculus.GRCm38.102) or human genome (GRCh38) using STAR (version 2.6.0a)[5] and then used for further analyses based on R version 4.1.0. Differential expression analysis was conducted using limma R package (version 3.48.3)[6,7]. The genes with a Benjamini–Hochberg adjusted P value < 0.05 and an absolute Log_2 (fold change) > 0 were identified as DEGs. Genes were ranked by Log_2 (fold change) and then employed to GSEA performed by clusterProfiler R package (version 4.2.2)[8]. The gene sets (Reactome, GO, Hallmarks, and KEGG) applied in GSEA were retrieved from the msigdbr R package (version 7.5.1)[9–11]. The gene sets with absolute normalized enrichment score (NES) higher than 1 and false discovery rate (qValue) lower than 0.05 were then identified as significantly enriched gene sets. Principal component analysis was performed using FactoMineR R package (version 2.9). Differential gene expression data of mPHs, mouse liver and HLOs are provided in Table S2, S4 and S6.

Estimation of liver cell type proportions

To estimate the cellular composition of the mouse liver samples, we performed single-cell deconvolution using MuSiC version 0.2.0[12] on raw bulk RNA-seq counts with the mouse liver scRNA-seq dataset as the reference[13]. The percentage of immune cells was determined by summing the contributions of NK, Kupffer, and B cells. Endothelial cells annotation refers to endothelial cells of the hepatic sinusoid. A t test and Benjamini–Hochberg multiple testing correction was used to test the significance of cell type composition between groups.

Mendelian randomization analysis

Mendelian randomization (MR) was used to test whether the activation of DNA damage response genes as a whole (rather than individually) might contribute to liver phenotypes. Using data from GTEx (dbGaP accession number phs000424.v8), we combined the expression of the constituent genes of either HepIRDS or DNA repair using principal component analysis (PCA) and selecting the first principal component (PC1). The orientation of PCs being arbitrary, we aligned them such that the median expression was positive to make interpretation easier (high PC values correspond to higher expression of most genes). Our interest was primarily with liver, however sample sizes are quite small (n = 208), so we also analyzed gene expression in blood (n = 670). To make results more comparable, we used the same weights for both blood and liver, namely the first eigenvector from the PCA in blood. We combined the genetic effect estimates at the summary statistics level using methods described previously[14], which is equivalent to combining the gene expression at the phenotype level and

then running a GWAS on the composite trait. Briefly, the PC weights are scaled to preserve the variance of the composite phenotype based on the phenotypic correlation:

$$\boldsymbol{v_{b1}} = \boldsymbol{w_{b1}} * \frac{\boldsymbol{1}}{\sqrt{\boldsymbol{w_{b1}^T} \cdot \boldsymbol{K} \cdot \boldsymbol{w_{b1}}}},$$

where $\boldsymbol{w_{b1}}$ is the unscaled vector of weights for PC1 (blood) and $\boldsymbol{K}$ is the phenotypic correlation matrix. The genetic effects can be calculated as

$$\boldsymbol{\beta} = \boldsymbol{B} \cdot \boldsymbol{v_{b1}},$$

where $\mathbf{B}$ is the matrix of gene expression in either blood or liver (both use the same weights from PC1 in blood). Finally, the variance of the effect can be calculated as

$$\boldsymbol{\sigma_i^2} = \boldsymbol{w_{b1}^T} \cdot \boldsymbol{\Sigma_i} \cdot \boldsymbol{K} \cdot \boldsymbol{\Sigma_i} \cdot \boldsymbol{w_{1b}},$$

where $\boldsymbol{\sigma_i}$ is the standard error of the composite effect of SNP i and $\boldsymbol{\Sigma_i}$ is a diagonal matrix with the standard error of the effect of SNP i on each gene.

As instrumental variables (IVs), we selected any variant which was a significant cis-eQTL for any of the genes in the set, as reported in GTEx v8[15] (https://www.gtexportal.org/home/). Rather than pruning for independence, these were combined using the PCA-based method proposed previously[16]. The LD structure for the PCA was estimated in the GTEx genetic data using Plink v1.90b6.21[17] (http://pngu.mgh.harvard.edu/purcell/plink/) and the PCA was only run on the subset of SNPs which were present in each individual outcome. PCs were selected to explain at least 99% of the variation, resulting in 3–20 PCs as IVs (Supplementary Table 7).

The summary statistics for all outcomes except liver fat fraction were obtained from the IEUGWAS catalog[18] using the ieugwasr R package (v0.1.5, REF: Hemani G (2023). ieugwasr: R Interface to the OpenGWAS Database API. R package version 0.1.5, https://github.com/MRCIEU/ieugwasr). We selected phenotypes based on relevance to liver health/disease and, where multiple GWASs were available, selected the one with the largest sample size. The genetic associations with liver fat fraction were calculated in the UK Biobank[19] (project 48020) using REGENIE[18] (v3.2.5). The UK Biobank is a large-scale population cohort in the UK of ~500,000 people aged 40-69 years old at the time of recruitment (2006–2010). Liver fat fraction was measured in ~30,000 participants by MRI (i.e., liver PDFF from the IDEAL protocol). The GWAS was performed on white European participants (based on continental ancestry from the Pan-UKB team. https://pan.ukbb.broadinstitute.org. 2020) and adjusted for sex, age, age^2, sex*age, sex*age^2 and the top 10 genetic principal components. MR results are provided in Table S7.

Total least squares (TLS) regression

Total least squares (TLS) regression was used in some cases to provide an estimate of the ratio between the changes induced by different interventions. This method was chosen as neither of the variables modeled could be considered error-free, which would result in regression attenuation. The slope of the regression would therefore be biased towards the null, which also means that the choice of dependent vs. independent variable would affect the estimated coefficient. TLS avoids this by minimizing the total distance, which accommodates errors in both variables.

Transcript-phenotype correlation in CC founder strains

Liver transcriptome and liver disease-related phenotypic traits were retrieved from Benegiamo et al[20]. PCA analyses were performed for gene sets of DNA repair, PARP family and HepIRDS using the FactoMineR R package (version 2.6)[21]. Pearson correlation was performed between PC1 or gene expression and disease phenotypic traits. P value were adjusted by Benjamini-Hochberg (BH). Transcript-phenotype correlation data are provided in Table S3.

The datasets used for correlation analyses between gene expression and clinical phenotypes are public available: the NASH-related phenotypic data (https://data.mendeley.com/datasets/dntgsyznzs/1) and liver transcriptome data (GSE201819) of seven inbred mouse strains, as previously published[20]. The liver proteome of C57BL/6J and CAST/EiJ strains are accessible at https://doi.org/10.17632/dntgsyznzs.1, as detailed in previous publication[20].

Human MASLD/MASH RNA-seq datasets

Human MASLD/MASH RNA-seq datasets are publicly available under the GEO of GSE162694, GSE135251 and GSE130970. The sample (subjects) were grouped based on the stage of fibrosis and Lowly expressed genes were filtered with the edgeR filter ByExpr function version 3.28.1[22]. Differential expression was performed using Limma-Voom with package version 3.42.2[7] on TMM-normalized counts computed with EdgeR calcNormFactors[22]. Differential expressed genes were determined with the contrast fibrosis ≥ 3 vs. fibrosis ≤ 1 accounted for sex using the design formula: ~fibro_group + sex. The significance threshold was set at 5% after Benjamini–Hochberg multiple testing correction.

TUNEL staining in mPH

mPHs were seeded in collagen-coated, glass bottom black plate (Cellvis) and allowed to attach for 5h before treatment with 1 mM doxorubicin ± 2.5 μM TLC-065 for 24h. Subsequently, cells were stained with TUNEL assay kit following the manufacturer's instruction (#ab66108, Abcam). Briefly, cells were fixed in 1% PFA at room temperature for 15 min and permeabilized with 0.1% Triton X-100 and 5% FBS for 10 min. The cells were then incubated in the DNA Labeling Solution for 60 min at 37 °C. DAPI was used for nucleus staining. Cells were imaged within 3 hours post-staining with a Leica SP8 inverted confocal microscope. DNA damage was quantified using CellProfiler[23] .

Fig. S1

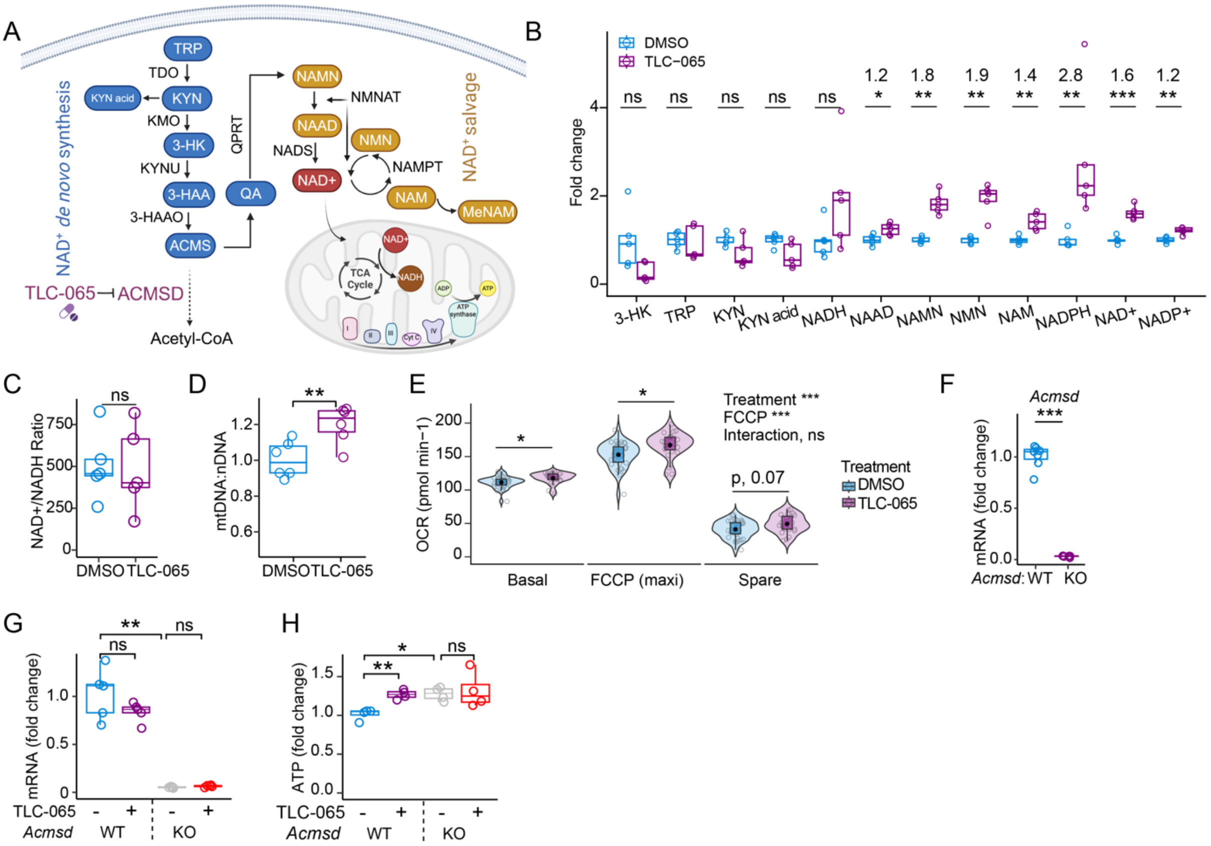

Fig. S1. Inhibiting ACMSD enhanced the NAD+ metabolome and improves mitochondrial respiration in hepatocytes. (**A**) Schematic of tryptophan-initiated NAD+ *de novo* synthesis. TRP, tryptophan; KYN, kynurenine; 3-HK, 3-OH kynurenine; 3-HAA, 3-OH, anthranilic acid; QA, quinolinic acid; NAMN, nicotinic acid mononucleotide; NAAD, nicotinic acid adenine dinucleotide; NMN, nicotinamide mononucleotide; NAM, nicotinamide; meNAM, methyl-NAM. Created with Biorender.com. (**B-D**) Fold changes of NAD^+ metabolome (n = 5) (**B**), NAD^+/NADH ratio (n = 5) (**C**), mtDNA:nDNA (n = 6) (**D**) in mPH treated with 0.5 µM TLC-065 or DMSO for 24h. (**E**) Oxygen consumption rate (OCR) in HuH-7 cells exposed to 10 µM TLC-065 for 24h (n = 34, DMSO; n = 24, TLC-065). (**F-H**) Fold changes of *Acmsd* expression (n =7 in **F**; n = 5 in **G**) and ATP levels (n = 4) (**H**) in *Acmsd* WT and KO mPH ± 0.1 µM TLC-065. *, $p < 0.05$; **, $p < 0.01$; ***, $p < 0.001$. Two-sided Student's t-test (B-D, F); Two-way ANOVA and Bonferroni multiple comparisons test (E, G-H).

Fig. S2

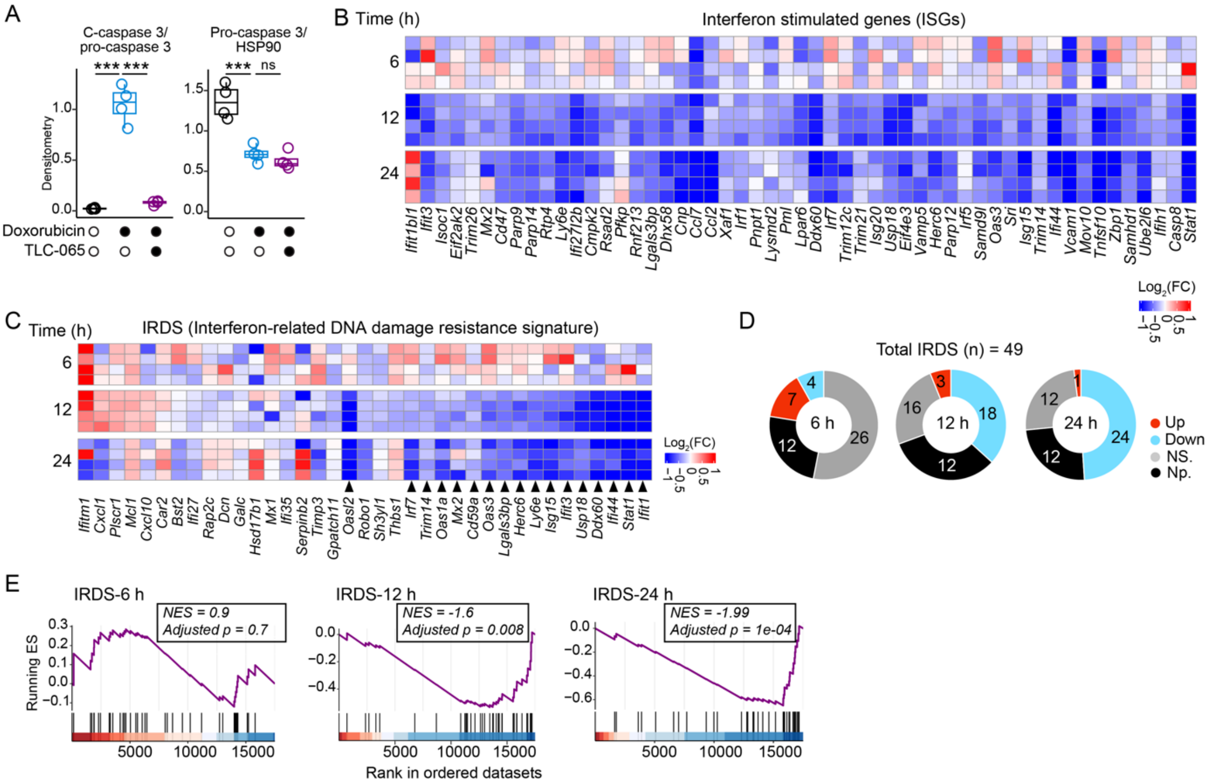

Fig. S2. Inhibiting ACMSD suppresses the innate immune response in mPHs. **(A)** Densitometry quantification of the western blot shown in Fig. 3E for cleaved-caspased 3 normalized to pro-caspase 3 and pro-caspase 3, normalized to HSP90 (n = 4). (**B-C**) Heatmap of the log_2-transformed FC of ISGs (**B**) and IRDS genes (**C**) in mPH treated with 0.5 μM TLC-065 for 6h, 12h, and 24h. Significantly downregulated IRDS overlapping at 12-h and 24-h are indicated by arrow heads. (**D**) Pie chart depicting the number of IRDS genes significantly up- or down-regulated by 0.5 μM TLC-065 at 6h, 12h, and 24h. NS., not significant; Np., Not present. (**E**) GSEA of the IRDS gene set after 6h, 12h, and 24h treatment with TLC-065. TLC-065 group was compared to their respective DMSO control at each timepoint. *, $p < 0.05$; **, $p < 0.01$; ***, $p < 0.001$; One-way ANOVA and Tukey's multiple comparisons test (A); Benjamini–Hochberg adjusted p values (C-D); FDR-corrected p values (E).

Fig. S3

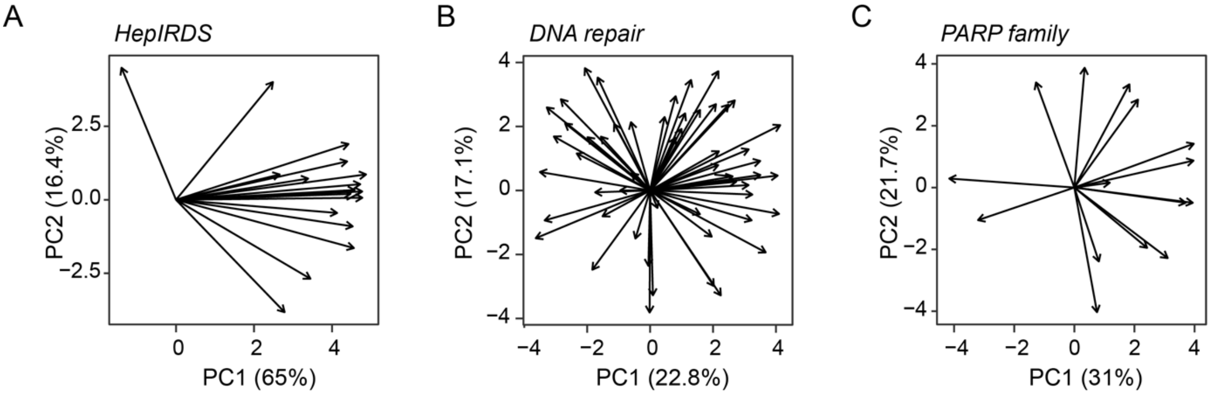

Fig. S3. Biplot showing the PCA analyses of DNA damage-related signatures in the liver transcriptome data of CC founder strains. Each gene is represented by one arrow, the coordinates of which correspond to that gene loading for PCs 1 and 2. The PC1 generalised the maximum variance of gene expression in each gene set and was therefore used in further correlation analyses between DNA damage-related gene sets and clinical phenotypes as shown in **Fig. 4B**. CC, seven genetically diverse Collaborative Cross founder strains. Arrows represent the genes of each gene set, with the length of arrows indicating the standard deviation of gene expression. The angles between arrows depict the expression correlations between genes.

Fig. S4

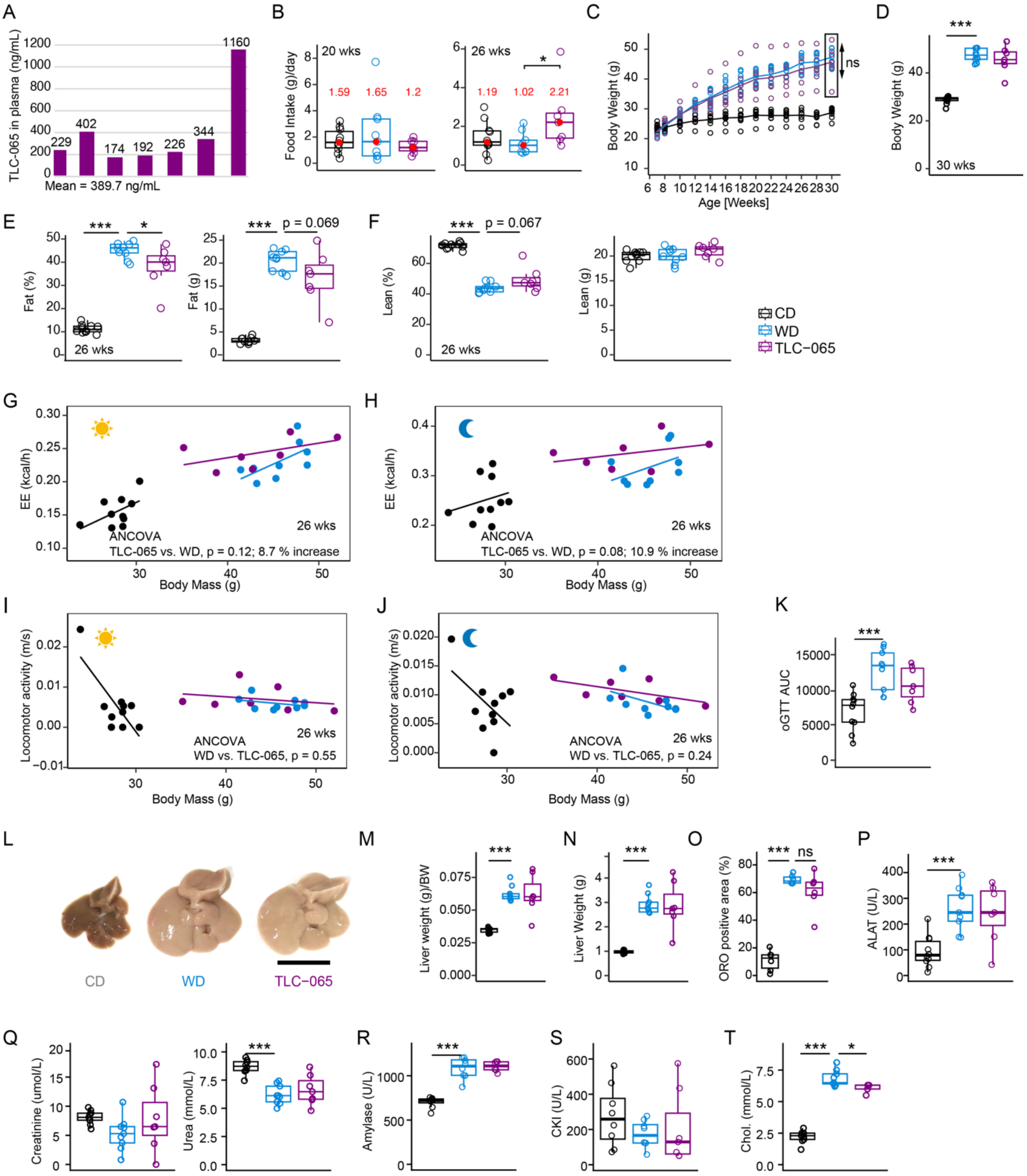

Fig. S4. TLC-065 treatment did not affect liver steatosis in WD/TN mice. (**A**) TLC-065 exposure analysis in plasma (n = 7). (**B**) A single-day food intake at 20 and 26 wks. Red dots represent the median values, which are labeled above each boxplot. (**C**) Body weight curves. The line represents the median. (**D-F**) Body weight at 30 wks (**D**), fat mass and its percentage at 26 wks (**E**), lean mass and its percentage at 26 wks (**F**). (**G-J**) Light (**G**) and dark (**H**) phases of energy expenditure (EE), light (**I**) and dark (**J**) phases of locomotor activity at 26 wks. Median of EE and of locomotor acitivity in light and dark phase was used for analysis. (**K**) Incremental glucose area under the curve (AUC) during oGTT test in Fig. 5B. (**L**) Representative gross liver morphology of 30 wks mice. Scale bar, 2 cm. (**M-O**) Ratio of liver weight to body weight (**M**), liver weight (**N**), and percentage of ORO^{+} area of liver sections (**O**). (**P-T**) plasma levels of ALAT (**P**), creatinine and urea (**Q**), amylase (**R**), creatine kinase (CKI) (**S**) and total cholesterol (Chol.) (**T**). CD n=10, WD n = 9, TLC-065 n = 7 (B-N); n = 6 for all groups (O); CD n= 8-10, WD n = 8-

9, TLC-065 n = 6-7 (P-T). *, p < 0.05; **, p < 0.01; ***, p < 0.001. One-way ANOVA and Tukey's multiple comparisons test (B, D-F, K, M-T). ANCOVA (G-J).

Fig. S5

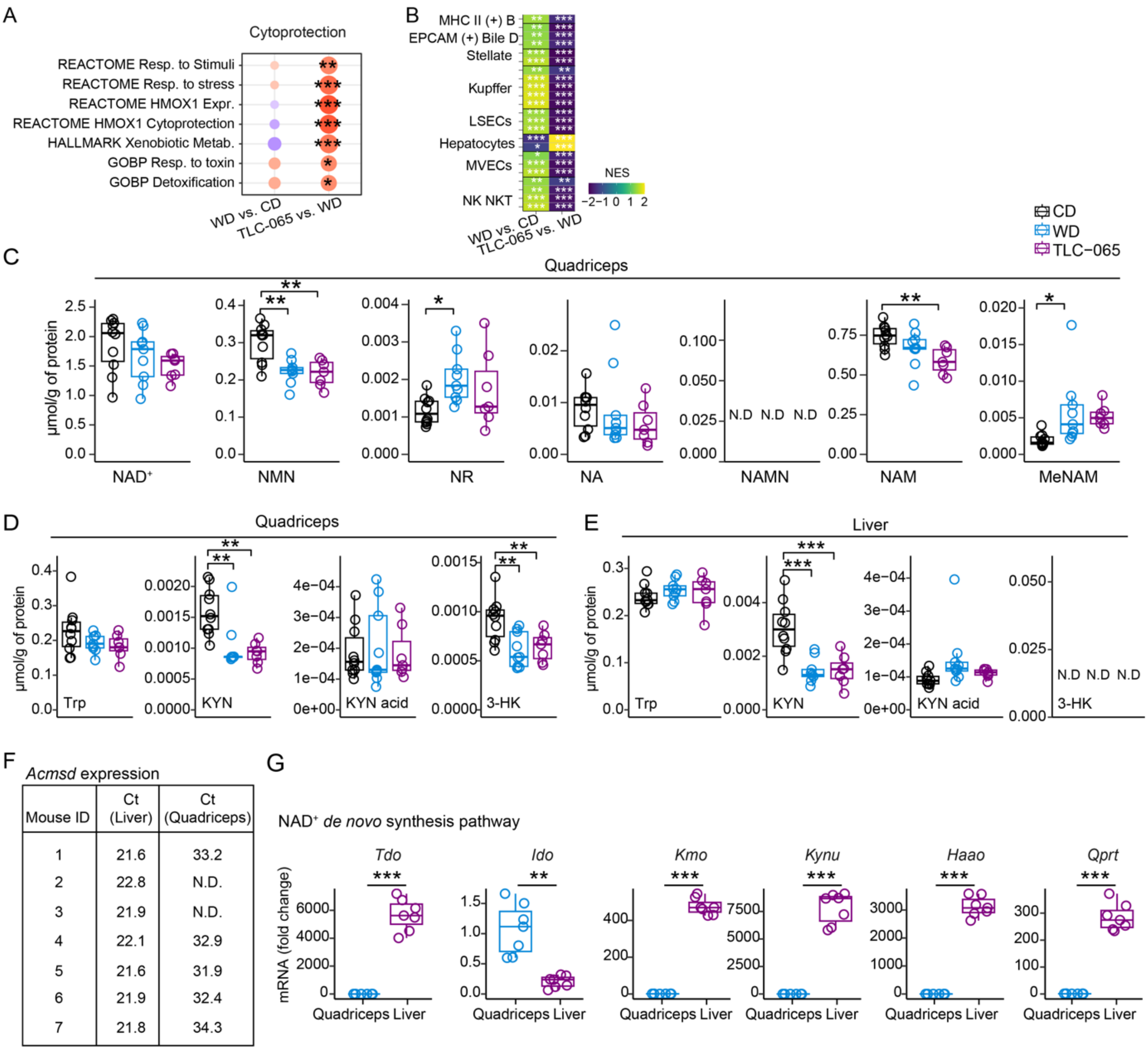

Acmsd expression

Mouse ID	Ct (Liver)	Ct (Quadriceps)
1	21.6	33.2
2	22.8	N.D.
3	21.9	N.D.
4	22.1	32.9
5	21.6	31.9
6	21.9	32.4
7	21.8	34.3

Fig. S5. TLC-065 suppresses liver cell population shifts and selectively enhances the liver metabolome in WD/TN mice. (**A**) GSEA of representative gene sets involved in cytoprotective processes. (**B**) GSEA of liver-specific cell types in the comparisons of WD vs. CD and of TLC-065 vs. WD. Every row represents a gene set for a cell type sub-population. n = 6, CD; n = 6, WD; n = 4, TLC-065. (**C-E**) NAD+ metabolome in quadriceps (**C**), kynurenine-related metabolites in quadriceps (**D**) and liver (**E**). Protein abundance was used for normalization. N.D. not dectected. (CD n = 10, WD n = 9, TLC-065 n = 7). (**F-G**) Expression of *Acmsd* (**F**) and other genes (**G**) in the NAD⁺ de novo synthesis pathway in liver and quadriceps. (n = 7) *, p < 0.05; **, p < 0.01; ***, p < 0.001. FDR-corrected p values (A-B). One-way ANOVA and Tukey's multiple comparisons test (C-E, G).

Fig. S6

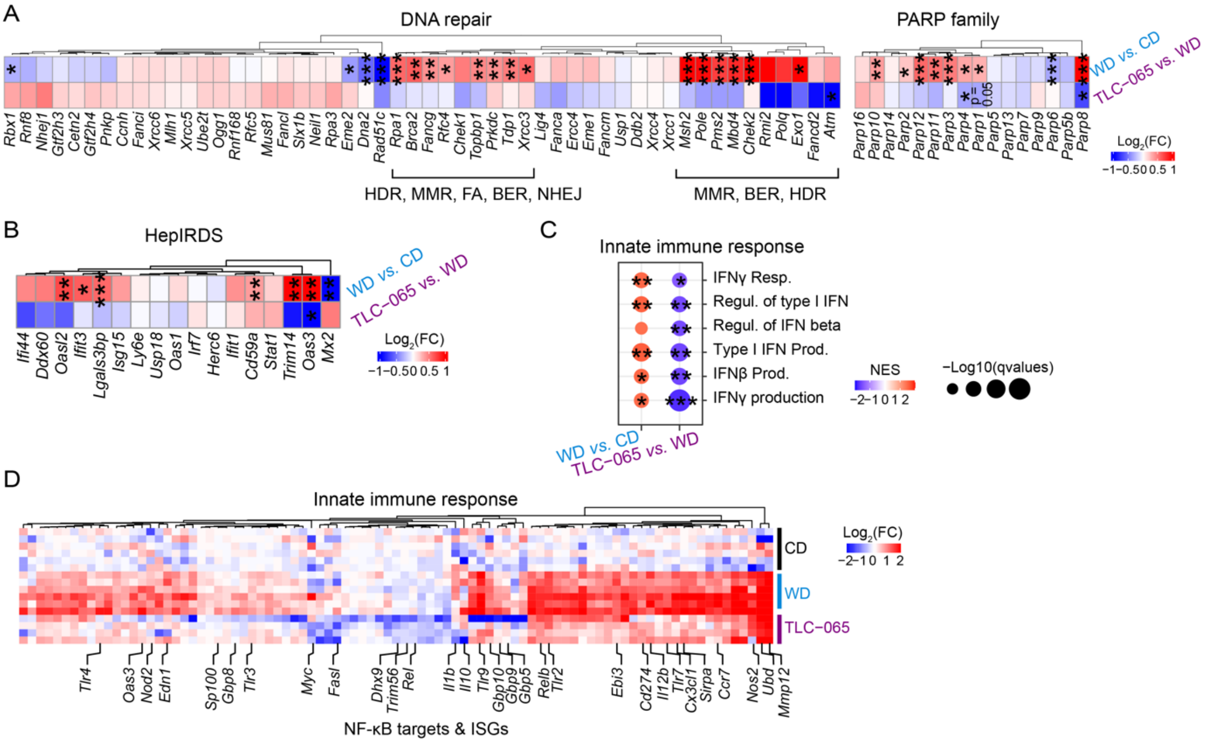

Fig. S6. TLC-065 counteracts the upregulation of the DNA repair and innate immune response in WD/TN mice. (**A-B**) Log2-transformed FC of DNA repair genes, the PARPs (**A**) and the HepIRDS genes (**B**). (**C**) Significantly enriched innate immune pathways determined by GSEA in WD/TN (WD vs. CD) and in TLC-065 treated mice (TLC-065 vs. WD). (**D**) Log2-transformed FC of core genes that contribute to the enrichment of the gene sets listed in **(C).** *, $p < 0.05$; **, $p < 0.01$; ***, $p < 0.001$; Benjamini–Hochberg adjusted p values (A-B). FDR-corrected p values (q values) (C).

Fig. S7

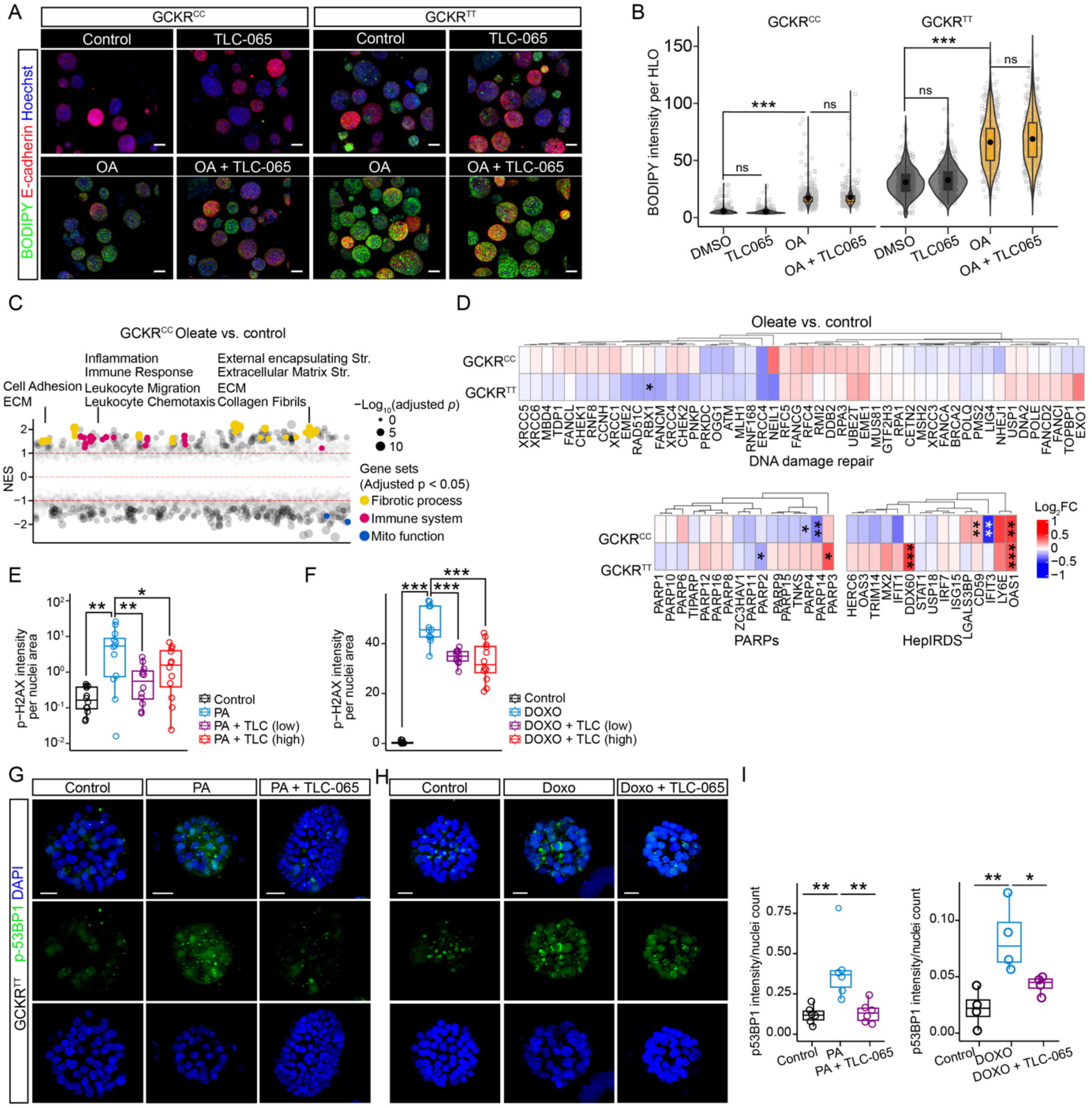

Fig. S7. ACMSD inhibition reduces DNA damage without affecting fat accumulation in HLO models. (**A**) Representative images of GCKR^{CC} and GCKR^{TT}-HLOs subjected to a 3-day regimen of oleate supplementation (OA, 300 μM), followed by 10 μM TLC-065 treatment. Images were stained with BODIPY for fat accumulation (green), Hoechst33342 for nuclei (blue) and E-cadherin for cell membranes (magenta). Scale bars, 70 μm. (**B**) Quantification of BODIPY intensity per HLO (n = 587)**.** (**C**) Manhattan plot showing GSEA for gene sets affected by oleate in GCKR^{CC} sHLO. Significant gene sets related to fibrosis, immune system and mitochondrial function are colored in yellow, red and blue. (**D**) Log$_2$-transformed FC of DNA repair genes, PARPs and HepIRDS genes in GCKR^{TT} or GCKR^{CC} under oleate treatment (oleate vs. control). (**E-F**) Quantification of p-H2A.X (Ser139) intensity in GCKR^{TT} upon 300 μM palmitate (PA) for 48h (n = 12) (**E**) or upon 5 μM doxorubicin (Doxo) for 24h (n = 12) (**F**). TLC (high): 10 μM; TLC (low): 1 μM. p-H2A.X intensity was normalized to nuclei area in comparisions. (**G-H**) Representative images of DNA damage signal p-53BP1 Ser1778 in GCKR^{TT} HLOs exposed to 300 μM palmitate (PA) for 48h (**G**) or to 5 μM doxorubicin for 24h (**H**) ± 10 μM TLC-065. p-53BP1 (ser1778) for DNA damage (green) and DAPI for nuclei (blue). Scale bars: 20 μm control (G); 30 μm PA and PA + TLC-065 (G); 20 μm (H). (**I**) Quantification of p-53BP1 intensity normalized to nuclei count (n = 6 for PA groups, n= 4 for Doxo groups). *, p < 0.05; **, p < 0.01; ***, p < 0.001; One-way ANOVA and Tukey's multiple comparisons test (B, E-F, I). Benjamini–Hochberg adjusted p values (D), FDR-corrected p values (C).

Table S8

Sequence	Primer	Gene	Experiment	Species
TGTCTTCAGCAACTGTGAGAGGA	fwd	Trib3	qPCR	mouse
CAGTCATCACGCAGGCATC	rev	Trib3	qPCR	mouse
CGGAACCTGAGGAGAGAGTG	fwd	Chop	qPCR	mouse
CGTTTCCTGGGGATGAGATA	rev	Chop	qPCR	mouse
ACTTGGGGACCACCTATTCCT	fwd	Grp78	qPCR	mouse
ATCGCCAATCAGACGCTCC	rev	Grp78	qPCR	mouse
CTCGTAGGAATGCCATCGGA	fwd	Acmsd	qPCR	mouse
CTGGGGTCAGACGGATTGTC	rev	Acmsd	qPCR	mouse
AGTGTGGCTCTTTTGCTCCA	fwd	Ddx60	qPCR	mouse
ACTCGCACCACTTTTTCATTTT	rev	Ddx60	qPCR	mouse
CAGGAGTCCCTGATTTGCGT	fwd	Usp18	qPCR	mouse
CAAGGCATCCTCCAGGGTTT	rev	Usp18	qPCR	mouse
TCTGCTCTGCTGAAAACCCA	fwd	Ifit1	qPCR	mouse
CACCATCAGCATTCTCTCCCAT	rev	Ifit1	qPCR	mouse
AGACTCTTCACACAGACTTCCG	fwd	Ifi44	qPCR	mouse
AACTTCTGCACACTCGCCTT	rev	Ifi44	qPCR	mouse
TAAGGTGGTGAAGGGAGGCT	fwd	Oasl2	qPCR	mouse
TGTTGTAGGCCAGGCTTCTG	rev	Oasl2	qPCR	mouse
GATCGCTTGCCCAACTCTTG	fwd	Stat1	qPCR	mouse
ACTGTGACATCCTTGGGCTG	rev	Stat1	qPCR	mouse
AGTACCGAAAGGCCAGAACC	fwd	Herc6	qPCR	mouse
TGTTCCTTCCCACAGCTCAC	rev	Herc6	qPCR	mouse
GGCTACCGTGTACGCATCT	fwd	Oas3	qPCR	mouse
CTTCACACAGCGGCCTTTACC	rev	Oas3	qPCR	mouse
TTCTGAACTGCTCAGCCCAC	fwd	Ifit3	qPCR	mouse
TCCCGGTTGACCTCACTCAT	rev	Ifit3	qPCR	mouse
TCTGACTGTGAGAGCAAGCAG	fwd	Isg15	qPCR	mouse
ACCTTTAGGTCCCAGGCCATT	rev	Isg15	qPCR	mouse
TGACTCTAAGATTGCAGGGTTG	fwd	Cd59a	qPCR	mouse
CTCATTGTGCTGGGCTACCA	rev	Cd59a	qPCR	mouse
GTCGCCTATTCACCAGGCTC	fwd	Mx2	qPCR	mouse
TCGTCCACGGTACTGCTTTT	rev	Mx2	qPCR	mouse
TTGCTCACTGTCTTTGCCTG	fwd	Oas1a	qPCR	mouse
GGATCACAGGCCTGGCTTT	rev	Oas1a	qPCR	mouse
GTCCGGAGCACAGCGAAC	fwd	Trim14	qPCR	mouse
CCTGGATTAGCTTCTGCACAC	rev	Trim14	qPCR	mouse
CCAGCTCTCACCGAGCG	fwd	Irf7	qPCR	mouse
GTTCTTACTGCTGGGGCCAT	rev	Irf7	qPCR	mouse

Supplementary references

1. Quiros PM, Goyal A, Jha P, Auwerx J. Analysis of mtDNA/nDNA Ratio in Mice. Curr Protoc Mouse Biology. 2017;7: 47–54.

2. Velpen V, Rosenberg N, Maillard V, Teav T, Chatton J, Gallart-Ayala H, et al. Sex-specific alterations in NAD+ metabolism in 3xTg Alzheimer's disease mouse brain assessed by quantitative targeted LC-MS. J Neurochem. 2021;159: 378–388. doi:10.1111/jnc.15362

3. Medina J, Velpen V van der, Teav T, Guitton Y, Gallart-Ayala H, Ivanisevic J. Single-Step Extraction Coupled with Targeted HILIC-MS/MS Approach for Comprehensive Analysis of Human Plasma Lipidome and Polar Metabolome. Metabolites. 2020;10: 495.

4. Chen Y, Chen Y, Shi C, Huang Z, Zhang Y, Li S, et al. SOAPnuke: A MapReduce Acceleration supported Software for integrated Quality Control and Preprocessing of High-Throughput Sequencing Data. GigaScience. 2017;7: gix120.

5. Dobin A, Davis CA, Schlesinger F, Drenkow J, Zaleski C, Jha S, et al. STAR: ultrafast universal RNA-seq aligner. Bioinformatics. 2013;29: 15–21.

6. Law CW, Chen Y, Shi W, Smyth GK. voom: precision weights unlock linear model analysis tools for RNA-seq read counts. Genome Biol. 2014;15: R29.

7. Ritchie ME, Phipson B, Wu D, Hu Y, Law CW, Shi W, et al. limma powers differential expression analyses for RNA-sequencing and microarray studies. Nucleic Acids Res. 2015;43: e47–e47.

8. Yu G, Wang L-G, Han Y, He Q-Y. clusterProfiler: an R Package for Comparing Biological Themes Among Gene Clusters. OMICS: A J Integr Biol. 2012;16: 284–287.

9. Liberzon A, Subramanian A, Pinchback R, Thorvaldsdóttir H, Tamayo P, Mesirov JP. Molecular signatures database (MSigDB) 3.0. Bioinformatics. 2011;27: 1739–1740.

10. Subramanian A, Tamayo P, Mootha VK, Mukherjee S, Ebert BL, Gillette MA, et al. Gene set enrichment analysis: A knowledge-based approach for interpreting genome-wide expression profiles. Proc Natl Acad Sci. 2005;102: 15545–15550.

11. Liberzon A, Birger C, Thorvaldsdóttir H, Ghandi M, Mesirov JP, Tamayo P. The Molecular Signatures Database Hallmark Gene Set Collection. Cell Syst. 2015;1: 417–425.

12. Wang X, Park J, Susztak K, Zhang NR, Li M. Bulk tissue cell type deconvolution with multi-subject single-cell expression reference. Nat Commun. 2019;10: 380.

13. Schaum N, Karkanias J, Neff NF, May AP, Quake SR, Wyss-Coray T, et al. Single-cell transcriptomics of 20 mouse organs creates a Tabula Muris. Nature. 2018;562: 367–372.

14. Sulc J, Sonrel A, Mounier N, Auwerx C, Marouli E, Darrous L, et al. Composite trait Mendelian randomization reveals distinct metabolic and lifestyle consequences of differences in body shape. Commun Biol. 2021;4: 1064.

15. Lonsdale J, Thomas J, Salvatore M, Phillips R, Lo E, Shad S, et al. The Genotype-Tissue Expression (GTEx) project. Nat Genet. 2013;45: 580–585. 16. Burgess S, Zuber V, Valdes-Marquez E, Sun BB, Hopewell JC. Mendelian randomization with fine-mapped

genetic data: Choosing from large numbers of correlated instrumental variables. Genet Epidemiology. 2017;41: 714–725.

17. Purcell S, Neale B, Todd-Brown K, Thomas L, Ferreira MAR, Bender D, et al. PLINK: A Tool Set for Whole-Genome Association and Population-Based Linkage Analyses. Am J Hum Genet. 2007;81: 559–575.

18. Elsworth B, Lyon M, Alexander T, Liu Y, Matthews P, Hallett J, et al. The MRC IEU OpenGWAS data infrastructure. Biorxiv. 2020; 2020.08.10.244293.

19. Sudlow C, Gallacher J, Allen N, Beral V, Burton P, Danesh J, et al. UK Biobank: An Open Access Resource for Identifying the Causes of a Wide Range of Complex Diseases of Middle and Old Age. PLoS Med. 2015;12: e1001779.

20. Benegiamo G, Alvensleben GVG von, Rodríguez-López S, Goeminne LJE, Bachmann AM, Morel J-D, et al. The genetic background shapes the susceptibility to mitochondrial dysfunction and NASH progression. J Exp Med. 2023;220: e20221738.

21. Lê S, Josse J, Husson F. FactoMineR : An R Package for Multivariate Analysis. J Stat Softw. 2008;25.

22. Robinson MD, McCarthy DJ, Smyth GK. edgeR: a Bioconductor package for differential expression analysis of digital gene expression data. Bioinformatics. 2010;26: 139–140.

23. Stirling DR, Swain-Bowden MJ, Lucas AM, Carpenter AE, Cimini BA, Goodman A. CellProfiler 4: improvements in speed, utility and usability. BMC Bioinform. 2021;22: 433.
